# Protein Kinase A Activity Is Necessary for Fission and Fusion of Golgi to Endoplasmic Reticulum Retrograde Tubules

**DOI:** 10.1371/journal.pone.0135260

**Published:** 2015-08-10

**Authors:** María J. Tenorio, Charlotte Luchsinger, Gonzalo A. Mardones

**Affiliations:** Instituto de Fisiología, Facultad de Medicina, and Centro Interdisciplinario de Estudios del Sistema Nerviso, Universidad Austral de Chile, Valdivia, Chile; Institut Curie, FRANCE

## Abstract

It is becoming increasingly accepted that together with vesicles, tubules play a major role in the transfer of cargo between different cellular compartments. In contrast to our understanding of the molecular mechanisms of vesicular transport, little is known about tubular transport. How signal transduction molecules regulate these two modes of membrane transport processes is also poorly understood. In this study we investigated whether protein kinase A (PKA) activity regulates the retrograde, tubular transport of Golgi matrix proteins from the Golgi to the endoplasmic reticulum (ER). We found that Golgi-to-ER retrograde transport of the Golgi matrix proteins giantin, GM130, GRASP55, GRASP65, and p115 was impaired in the presence of PKA inhibitors. In addition, we unexpectedly found accumulation of tubules containing both Golgi matrix proteins and resident Golgi transmembrane proteins. These tubules were still attached to the Golgi and were highly dynamic. Our data suggest that both fission and fusion of retrograde tubules are mechanisms regulated by PKA activity.

## Introduction

Membrane trafficking along the endocytic and secretory pathways is a highly regulated process. This regulation ensures that the associated cellular compartments fulfill their functions by maintaining their characteristic set of resident macromolecules. A central organelle at the crossroad of these pathways is the Golgi apparatus. In mammalian cells the Golgi comprises stacked, *cis* to *trans* cisternae organized in a perinuclear, ribbon-like structure, and a Golgi matrix. The membranes of the cisternae contain enzymes involved in post-translational modifications, as well as molecules related to the sorting of transported cargo to their sites of function. The Golgi matrix is composed of coiled-coil proteins that interact with cisternal transmembrane proteins and lipids, and provide adhesion of cisternae and tethers for docking of transport intermediates [1]. The coordinated anterograde and retrograde transfer of cargo molecules allows the *cis*-Golgi to distribute materials from and to the endoplasmic reticulum (ER), and the *trans*-Golgi to and from the plasma membrane, while preserving the characteristic structure of the Golgi. Initial electron microscopy studies suggested that the transfer of cargo is mediated by vesicular transport intermediates (i.e. vesicles; [2]). Although vesicles do have essential functions in transport [3], a different type of transport intermediates in the form of large pleiomorphic carriers (i.e. tubules) are responsible for moving the bulk of traffic between distant compartments, including the Golgi [4]. In any of these membrane transport events, two processes are intrinsic: the generation of transport intermediates from a donor compartment, and the fusion of transport intermediates with an acceptor compartment. The generation of transport intermediates involves a series of different mechanisms, such as the recruitment of cytosolic proteins, deformation of the lipid bilayer, association with the cytoskeleton, and ultimately the pinch-off from the membrane (i.e. fission; e.g., [5]). Similarly, the fusion of transport intermediates relies on a series of concerted events such as the correct delivery to the acceptor membrane through the cytoskeleton, and the tethering, docking and final fusion (e.g., [6, 7]). Despite a great deal of understanding of the molecular mechanisms of vesicular transport, less is known about tubular transport, in particular the regulation of fission and fusion of tubular carriers [8]. On the other hand, a regulatory role for signal transduction molecules in transport routes along both the endocytic and secretory pathways is becoming increasingly apparent. Several heterotrimeric G proteins have been implicated in different steps of membrane trafficking [9]. Second messengers and protein kinases have also been shown to influence various transport processes. For instance, several reports indicate that PKA regulates vesicle-mediated protein transport in the secretory [10–12], endocytic (e.g. [13, 14]), and Golgi-to-ER retrograde [15] pathways. The fact that the holoenzyme of PKA subtype-II is concentrated at both the *cis*- and *trans*-Golgi [16] points to additional functions in the control of transport activities at sites of vesicle and tubule formation. However, the targets of PKA activity are not well defined, and the processes regulated have not been characterized.

Previously, we reported that when cells are treated with the fungal metabolite brefeldin A (BFA), *cis* and medial Golgi matrix proteins move to ER exit sites by association with tubules [17]. Retrograde Golgi-to-ER tubule dynamics can be schematically viewed as the result of the processes of tubule formation, tubule elongation, tubule fission and tubule fusion. Currently, these processes are poorly characterized. In the present study, we addressed the question of whether this Golgi-to-ER retrograde transport of Golgi matrix proteins would be affected by PKA activity inhibition. We found that the Golgi-to-ER retrograde transport of Golgi matrix proteins was precluded in the presence of PKA inhibitors. Unexpectedly, we found the accumulation of tubules containing both Golgi matrix proteins and resident Golgi transmembrane proteins. These accumulated, retrograde tubules were attached to the Golgi and highly dynamic, and their tips were enriched in both Golgi matrix and transmembrane proteins. Our results show that both the fission and fusion of retrograde tubules are regulated by PKA activity.

## Experimental Procedures

### Cell culture

NRK (rat kidney fibroblasts), and HeLa (human epithelial) cells were obtained from the American Type Culture Collection (Manassas, VA). MDA-MB-231 (human breast adenocarcinoma), and MCF-7 (human breast adenocarcinoma) cells were kindly provided by P. Ehrenfeld (Department of Anatomy, Histology and Pathology, Universidad Austral de Chile). NRK and HeLa cells were maintained in DMEM (Life Technologies). MDA-MB-231 and MCF-7 cells were maintained in DMEM-F12 or MEM (Life Technologies), respectively. For all cell lines the media were supplemented with 10% heat-inactivated fetal bovine serum, 100 U/ml penicillin, and 100 μg/ml streptomycin (Life Technologies). MCF-7 cells were further supplemented with 10 μg/ml insulin (Sigma-Aldrich).

### Cell reagents and Antibodies

BFA, cycloheximide, and cytochalasin D were from Sigma-Aldrich. Calphostin C, chelerythrine, N-[2-((*p*-Bromocinnamyl) amino) ethyl]-5-isoquinolinesulfonamide (H-89), 4-cyano-3-methylisoquinoline, (9*R*,10*S*,12*S*)-2,3,9,10,11,12-Hexahydro-10-hydroxy-9-methyl-1-oxo-9,12-epoxy-1*H*-diindolo[1,2,3-*fg*:3',2',1'-*kl*]pyrrolo[3,4-*i*][1,6]benzodiazocine-10-carboxylic acid, hexyl ester (KT5720), myristoylated protein kinase A inhibitor amide 14–22 (myr-PKI-A), myristoylated protein kinase C inhibitor 20–28 (myr-PKI-C), nocodazole, and *R*_p_-adenosine 3′,5′-cyclic monophosphorothioate (*R*_p_-cAMPS) were from Calbiochem. N^6^-Monobutyryladenosine-3', 5'-cyclic monophosphate (6-MB-cAMP) was from BIOLOG Life Science Institute (Bremen, Germany). We used monoclonal antibodies to the following proteins: GM130, GRASP55 and GS28 (BD Biosciences); rat α-mannosidase II (Man-II; Covance), giantin [18], β-COP (clone M3A5; [19]), β-tubulin (GE Healthcare), and GalNAc-transferase 1 and GalNAc-transferase 2 [20] (from U. Mandel, University of Copenhagen, Nørre Alle, Denmark). We used polyclonal antibodies to the following proteins: human Man-II (Chemicon International), rat Man-II (Carbohydrate Research Center, University of Georgia, Athens, GA), p115 [21], GM130 [22], GRASP65 (Santa Cruz Biotechnology), and Sec23A (Affinity Bioreagents). DAPI (4',6-Diamidino-2-Phenylindole), tetramethylrhodamine-conjugated phalloidin, and the following fluorescently labeled antibodies were from Life Technologies: Alexa Fluor-488- or -594-conjugated donkey anti mouse IgG, Alexa Fluor-488- or -594-conjugated donkey anti rabbit IgG, and Alexa Fluor-488-conjugated donkey anti goat IgG. All primary antibodies were used at a dilution 1/200; all secondary antibodies were used at a dilution 1/1000.

### Immunofluorescence Microscopy

Samples for immunofluorescence analysis were prepared as described previously [23]. Fixation of cells with methanol or 4% paraformaldehyde were performed when primary antibody reactivity demanded, and gave the same results; however tubular profiles were best preserved in cells fixed with paraformaldehyde. Wide-field fluorescence microscopy images were acquired with an Axiovert 200M microscope equipped with a PlanApochromat 63x oil immersion objective (NA 1.4; Carl Zeiss), using SlideBook acquisition software (Intelligent Imaging Innovations, Denver, CO) without deconvolution of the images, or with an AxioObserver.D1 microscope equipped with a PlanApo 63x oil immersion objective (NA 1.4), and an AxioCam MRm digital camera (Carl Zeiss), using similar settings as described previously [23]. Laser scanning confocal microscopy images were acquired with an Olympus FluoView FV1000 scanning unit fitted on an inverted Olympus IX81 microscope equipped with a PlanApo 60x oil immersion objective (NA 1.4; Olympus, Melville, NY), using similar settings as described previously [24]. For quantification of phenotypes, two hundred cells were counted for each experiment.

### PKA Activity Assay

PKA activity was assessed using a non-radioactive protein kinase assay kit (Calbiochem; cat # 539484), according to the manufacturer's instructions.

### Recombinant cDNA Constructs and Transfection

We used plasmids encoding the following proteins tagged with the green fluorescent protein (GFP) or spectral variants of GFP: GFP-Rab6A [25]; GFP-GRASP55 and GFP-GRASP65 [26, 27]; CFP-GM130 and YFP-GM130 [28]; p115–YFP [29]; GalT-CFP and GalT-YFP, coding for the transmembrane domain of β-1,4-galactosyltransferase fused to CFP or YFP at the luminal domain [30, 31]; and Sec13-YFP [32]. Transient transfections in NRK cells were performed using Lipofectamin 2000 (Life Technologies), according to the manufacturer's instructions. NRK cells stably expressing GFP-GRASP55 were produced by selection for Geniticin (G418) resistance (at 500 μg/ml; Invitrogen) expressed by the appropriate plasmid.

### Time-Lapse Microscopy

Time-lapse fluorescence imaging was as described [17]. Briefly, live NRK cells were held at 37°C on a microscope heater stage (Warner Instruments, Hamden, CT), and images were acquired with an inverted Olympus IX81 microscope (Olympus America) equipped with a PlanApo 60x oil immersion objective (NA 1.4; Olympus America), and an attached Hamamatsu ORCA IIER monochromatic charge-coupled device camera (Hamamatsu, Bridgewater, NJ), using SlideBook acquisition software (Intelligent Imaging Innovations). Filter sets were from Chroma Technology (Brattleboro, VT). Images were processed and converted to Quicktime movies with MetaMorph software (Molecular Devices, Sunnyvale, CA). To prepare figures, single frames were processed with Adobe Photoshop CS3 (Adobe Systems, Mountain View, CA).

## Results and Discussion

### Retrograde tubules are accumulated upon treatment with H-89

**Fig 1:**
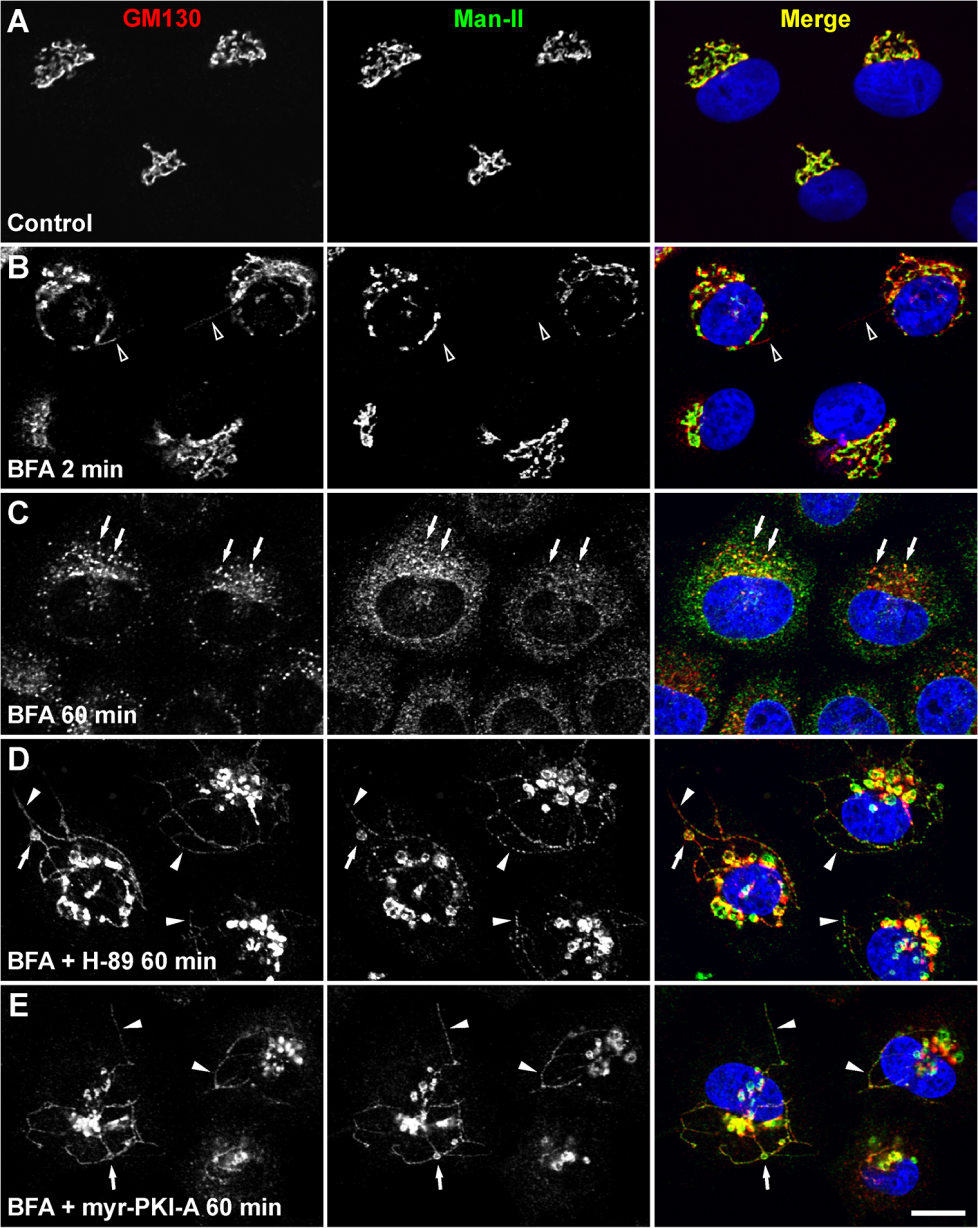
Retrograde tubules accumulate upon PKA inhibition. NRK cells were left untreated (A) or treated with 5 μg/ml BFA (B-C), or with BFA in conjunction with either 30 μM H-89 (D) or 100 μM myr-PKI-A (E), for the time indicated. Cells were fixed, permeabilized, immunolabeled with mouse monoclonal antibody to GM130 and rabbit antibody to α-mannosidase II (Man-II), followed by Alexa-594-conjugated donkey anti-mouse IgG (red channel), and Alexa-488-conjugated donkey anti-rabbit IgG (green channel). Nuclei were stained with DAPI (blue channel). Stained cells were examined by confocal fluorescence microscopy. Merging of the images in the red, green, and blue channels generated the third image on each row; yellow indicates overlapping localization of the green and red channels. In B, arrowheads indicate the emergence of tubules containing GM130, but devoid of Man-II. In C, arrows indicate colocalization at ER exit sites. In D and E, arrowheads indicate colocalization at accumulated tubules, and arrows indicate colocalization at swollen portions of tubules. Bar, 10 μm.

During BFA treatment *cis*- and medial-Golgi resident transmembrane proteins are redistributed to the ER via tubules [33]. In HeLa cells the retrograde transport of these Golgi proteins is blocked by the protein kinase inhibitor H-89 [34], suggesting that protein phosphorylation plays a role in tubulation at the Golgi-ER interface. During BFA treatment *cis*- and medial Golgi matrix proteins also redistribute associated with retrograde tubules, but to ER exit sites [17]. First, we evaluated if the blockage of retrograde transport by H-89 also occurs for *cis*- and medial Golgi matrix proteins. For these analyses we used NRK cells. As expected, after 2 min of BFA treatment (5 μg/ml), Golgi matrix proteins lead the redistribution in tubules, as shown by immunolocalization of the Golgi matrix protein GM130, and the resident transmembrane protein α-mannosidase II (Man-II) (Fig 1B; [17]). After 60 min of BFA treatment, there is a complete redistribution of both Man-II into the ER [35], and GM130 at the vicinity of ER exit sites, in addition to some colocalization at these puncta (Fig 1C; [17, 32, 36]). Co-incubation for 60 min with BFA and 30 μM H-89 resulted in complete blockage of the redistribution of GM130 and Man-II, similar to what has been shown in HeLa cells for GM130 and the type-II Golgi membrane protein GPP130 [34] (Fig 1D). Surprisingly, and in contrast to what was reported for HeLa cells [34], in ~35% of NRK cells we saw an accumulation of tubules emanating from the Golgi, tubules positive for both GM130 and Man-II (Fig 1D). Treatment with BFA in conjunction with H-89 did not block β-COP dissociation from the Golgi and hence tubules were devoid of COPI (S1B Fig). Time-course experiments showed similar kinetics in the appearance of tubules as in cells treated with BFA alone (~2 min; S1B Fig, S2B Fig, and S3B Fig), suggesting that H-89 is not delaying the initiation in tubulation, thus tubule accumulation is likely to be a consequence of an inhibitory effect on the next steps in tubular retrograde transport.

Accumulated tubules curve and branch, but branching was less in extent compared to that observed during the time frame of high tubulation with BFA alone (S4A and S4B Fig). Three-dimensional reconstruction of optical sectioning showed complete continuity of tubules (S5 Fig). The number and size of accumulated tubules varied, from 1–11 tubules and ~3–20 μm, with the majority of the cells (~65% of tubule-containing cells) having 3–4 tubules ~10 μm long. Two other features were consistently observed: 1) the tip of some tubules were dilated (~10% of tubules; S6 Fig; see below), and 2) the central section of some tubules contained a distension or swelling (~3% of tubules; Fig 1D). After 2 h of coincubation with BFA and 30 μM H-89, inhibition of retrograde transport was still total (100% of cells), although there were fewer tubules accumulated (1 to 4 per cell), and in a smaller proportion of cells (~15% of cells). Longer co-incubations of up to three hours produced no additional changes in tubule accumulation, but cells started to round off (data not shown). Nevertheless, the treatment was completely reversible since washing out both BFA and H-89 restored normal cell appearance, even under protein synthesis inhibition (200 μg/ml cycloheximide), with the Golgi morphology indistinguishable from that of untreated cells (S7A Fig), and cells remained viable and able to grow for at least 72 more hours (data not shown). Recovery from co-incubation was faster: between 20 to 30 min after BFA and H-89 removal was sufficient to reach normal Golgi morphology, instead of 60 min after removal of BFA in cells treated with BFA alone (data not shown). We treated cells with 30 μM H-89 alone, and, importantly, we also observed accumulation of tubules that contained both GM130 and Man-II (S7B Fig); however the tubules were shorter (1–10 μm), and less in number per cell (1–4). Although this treatment led to the accumulation of tubules in only ~5% of the cells, this is consistent with the less frequent observation of retrograde tubules in untreated cells [37], and with the hypothesis that BFA amplifies a tubular, Golgi-to-ER transport pathway [33, 38, 39].

### Retrograde tubules containing Golgi matrix proteins accumulate upon PKA activity inhibition

H-89 has been widely used as a potent PKA inhibitor [12, 15, 40], suggesting that the accumulation of BFA-induced tubules is associated with PKA activity [41]. To determine if the PKA activity is related to the accumulation of retrograde tubules, we used a panel of mechanistically diverse inhibitors of PKA. Treatment with BFA in conjunction with 30 μM 4-cyano-3-methylisoquinoline, 50 μM *R*_p_-cAMPS, 10 μM KT5720 (data not shown), or 100 μM myr-PKI-A (Fig 1E) produced a similar accumulation of tubules as in cells treated with BFA and H-89. However, myr-PKI-A produced accumulation of tubules in a fewer number of cells (~25%), with ~80% of cells showing inhibition of retrograde transport for both GM130 and Man-II (Fig 1E). This inhibition by myr-PKI-A on fewer cells was due presumably to lower permeability, since the accumulation of tubules occurred only after a minimum of 2 min of pre-incubation before BFA addition. Nevertheless, treatment with myr-PKI-A, as well as with the other inhibitors, resulted in similar effects as those observed with H-89, such as no impairment of redistribution of β-COP (S1C Fig, and data not shown), less branching of accumulated tubules (S4C Fig and data not shown), reversibility (S7C Fig and data not shown), and tubulation when incubated in the absence of BFA (S7D Fig and data not shown). The observations with this panel of structurally diverse inhibitors strongly argued in favor of a role for PKA activity in the accumulation of tubules. We did not conduct experiments using other treatments that interfere with PKA activity, such as RNAi or overexpression of dominant negative variants of PKA, because the prolonged time course of these experiments could result in adaptation of cells, and phenotypes could arise as a consequence of cumulative effects. In fact, RNAi of the regulatory, Golgi-localized RIIα subunit of PKA results in Golgi apparatus fragmentation [42]. Therefore, to obtain additional evidence that the effects on tubulation of H-89, as well as of the other inhibitors, could be the result of PKA inhibition, we used the cAMP analog 6-MB-cAMP. A number of cAMP analogs, such as 6-MB-cAMP, can specifically activate PKA [41, 43, 44]. Thus, the demonstration that these activators have effects opposite to those of inhibitors, such as H-89, has been suggested as appropriate to propose that PKA mediates the functions under investigation [41]. Indeed, pre-incubation with 6-MB-cAMP abolished the accumulation of tubules in cells treated with BFA and H-89, as well as in cells treated with BFA and either of the other (PKA) inhibitors, and in a fashion that was indistinguishable from the treatment of cells only with BFA (S7E Fig and data not shown).

**Fig 2:**
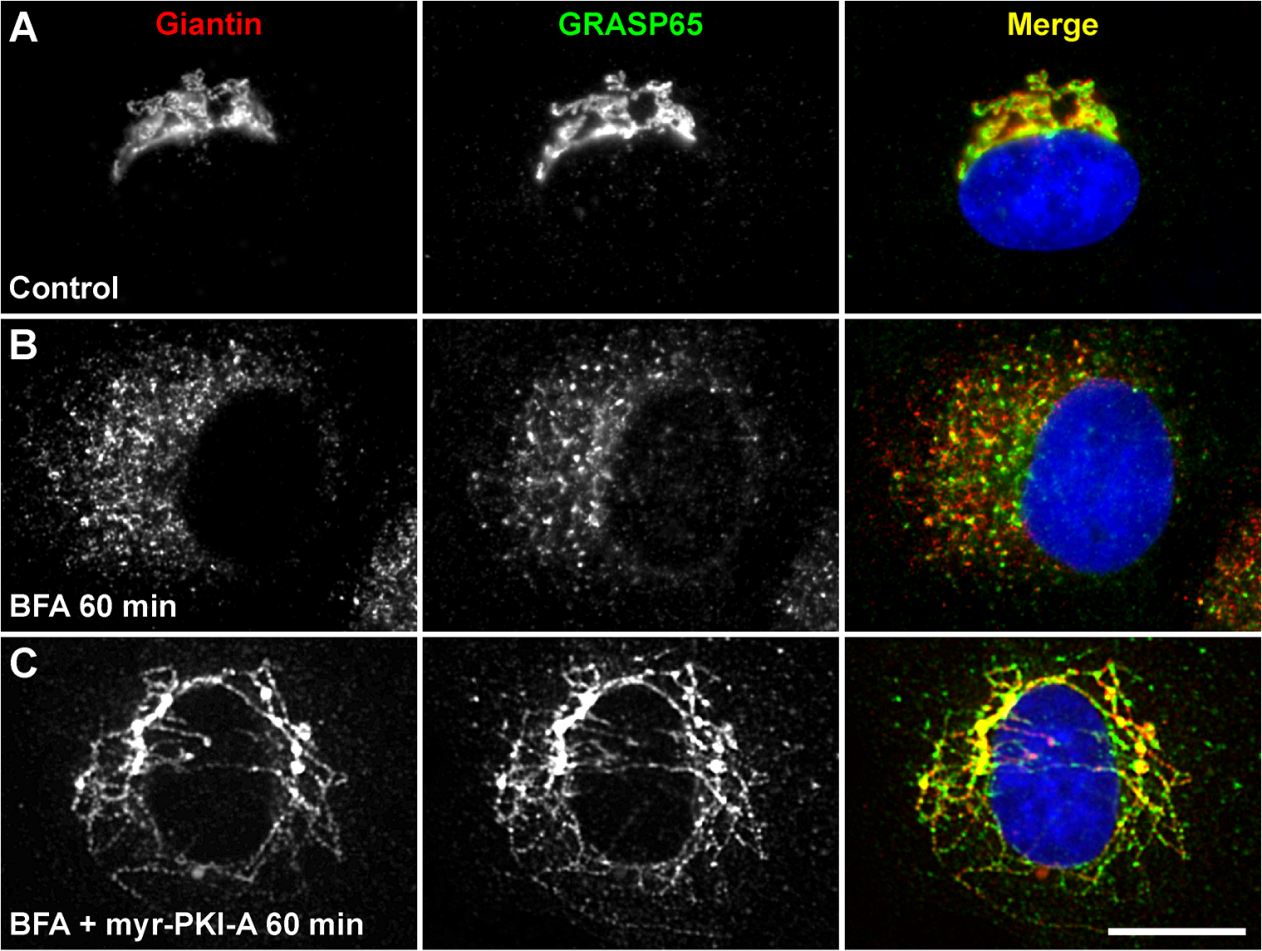
Giantin and GRASP65 colocalize in accumulated retrograde tubules. NRK cells were left untreated (A) or treated with 5 μg/ml BFA (B), or with BFA in conjunction with 100 μM myr-PKI-A (C), for the time indicated. Cells were fixed, permeabilized, immunolabeled with mouse monoclonal antibody to Giantin and rabbit antibody to GRASP65, followed by Alexa-594-conjugated donkey anti-mouse IgG (red channel), and Alexa-488-conjugated donkey anti-rabbit IgG (green channel). Nuclei were stained with DAPI (blue channel). Stained cells were examined by fluorescence microscopy. Merging of the images in the red, green, and blue channels generated the third image on each row; yellow indicates overlapping localization of the green and red channels. Bar, 10 μm.

**Fig 3:**
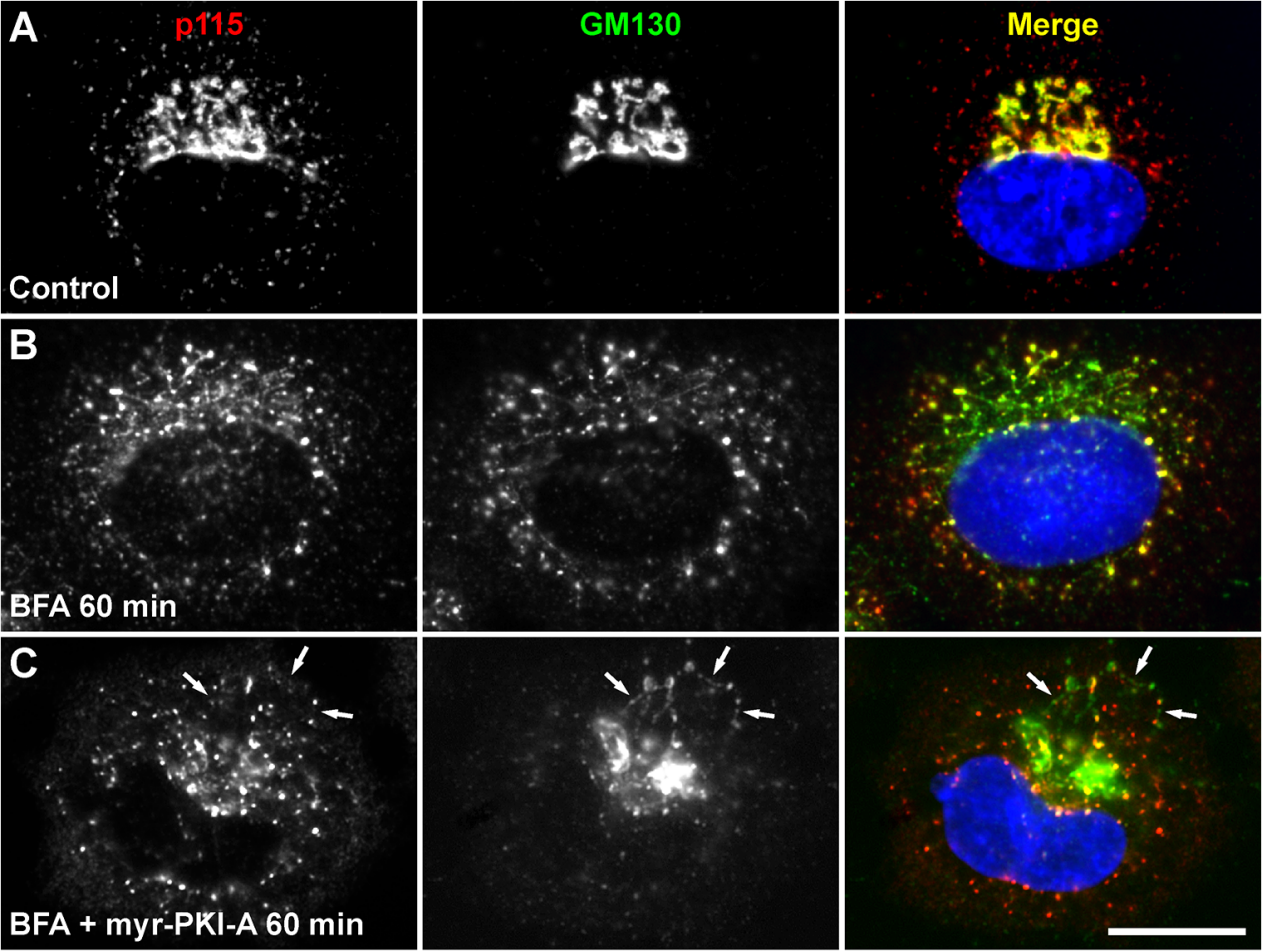
P115 and GM130 localizes distinctly in accumulated retrograde tubules. NRK cells were left untreated (A) or treated with 5 μg/ml BFA (B), or with BFA in conjunction with 100 μM myr-PKI-A (C), for the time indicated. Cells were fixed, permeabilized, immunolabeled with rabbit polyclonal antibody to p115 and mouse monoclonal antibody to GM130, followed by Alexa-594-conjugated donkey anti-rabbit IgG (red channel), and Alexa-488-conjugated donkey anti-mouse IgG (green channel). Nuclei were stained with DAPI (blue channel). Stained cells were examined by fluorescence microscopy. Merging of the images in the red, green, and blue channels generated the third image on each row; yellow indicates overlapping localization of the green and red channels. In C, arrows indicate colocalization in accumulated tubules. Bar, 10 μm.

Accumulated tubules showed association of other *cis*- and medial-Golgi proteins. The *cis*-Golgi matrix proteins giantin and GRASP65 (Fig 2C, S8A–S8C Fig and S9 Fig), as well as the medial-Golgi matrix protein GRASP55 (S6 Fig), and the transmembrane, Golgi resident proteins GalNAc-transferase 1 and GalNAc-transferase 2 (data not shown), localized well at accumulated tubules. However, a significant proportion of the *cis*-Golgi matrix protein p115 was enriched at peripheral puncta reminiscent of ER exit sites (Fig 3C, S10A–S10C Fig and S11 Fig), suggesting that p115 is less sensitive to the inhibition of retrograde transport. Other proteins known to function at the ER-Golgi interface were also found in accumulated tubules, such as GS28 of the SNARE family of proteins, or GFP-Rab6A (data not shown). Pre-incubation with 6-MB-cAMP also resulted in redistribution of all these additional set of Golgi proteins, in a manner indistinguishable to the treatment with BFA only (S8D Fig, S10D Fig and data not shown).

Golgi-to-ER retrograde tubules are associated with microtubule tracks [45]. Thus, as expected, the accumulation of tubules was reliant on microtubule integrity assessed by nocodazole treatment, but not affected by actin cytoskeleton poisoning with cytochalasin D (data not shown). Tubules emanating from the Golgi also accumulate in cells incubated at 15°C [46]. However, tubules accumulated at low temperature are devoid of Golgi matrix proteins [46]. Moreover, tubules accumulated at low temperature seem to mediate intra-Golgi transport [46], and appear to be mechanistically distinct to the tubulation induced by BFA [47]. Therefore, altogether, these data suggest that PKA activity is necessary for normal Golgi-to-ER tubular transport, and that both Golgi matrix and Golgi transmembrane proteins are equally present in accumulated tubules.

### Accumulation of retrograde tubules in NRK and HeLa cells has different sensitivities to H-89

**Fig 4:**
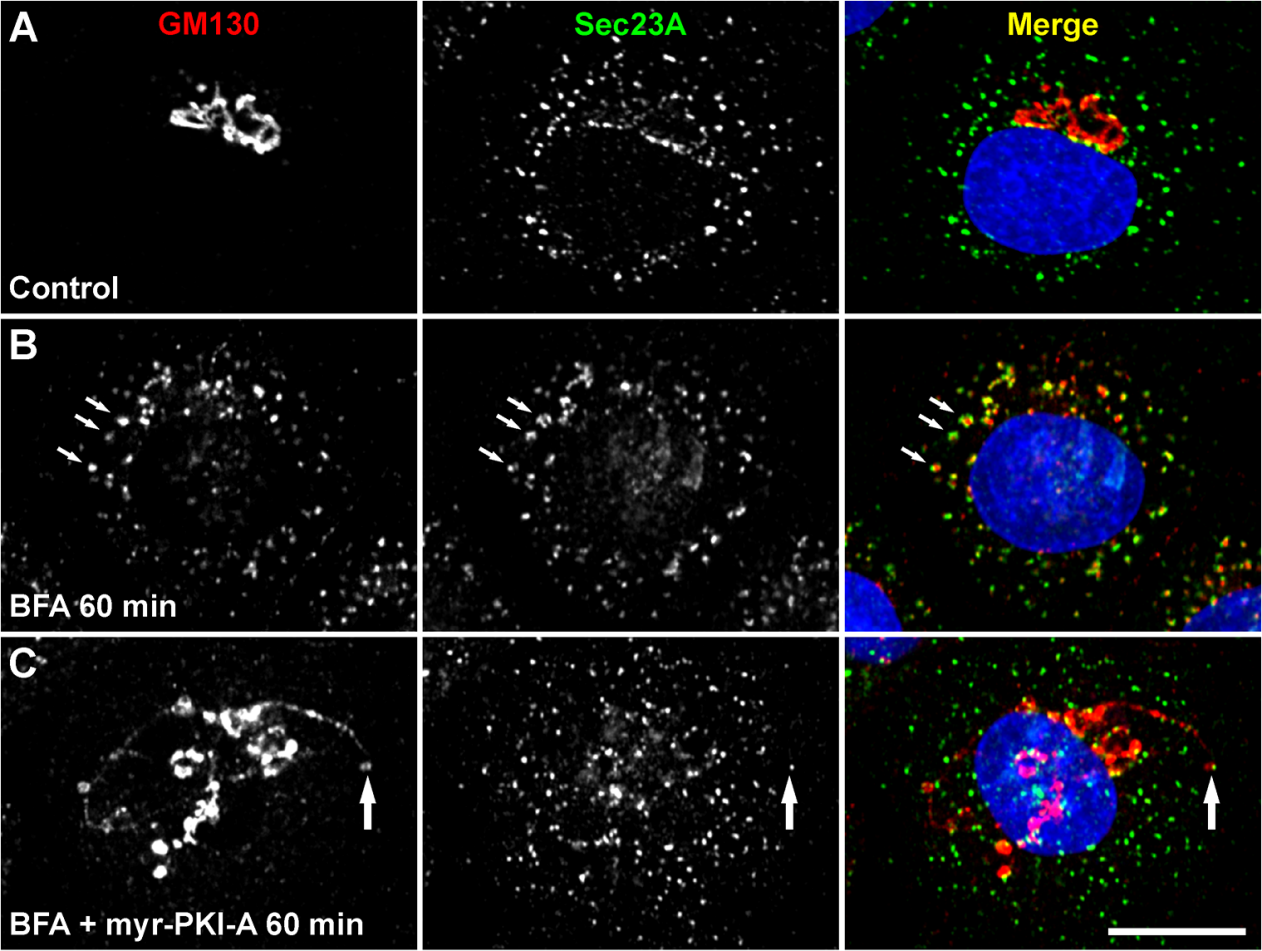
The tip of an accumulated tubule containing GM130 colocalizes with an ER exit site. NRK cells were left untreated (A) or treated with 5 μg/ml BFA (B), or with BFA in conjunction with 100 μM myr-PKI-A (C), for the time indicated. Cells were fixed, permeabilized, immunolabeled with mouse monoclonal antibody to GM130 and rabbit antibody to Sec23A, followed by Alexa-594-conjugated donkey anti-mouse IgG (red channel), and Alexa-488-conjugated donkey anti-rabbit IgG (green channel). Nuclei were stained with DAPI (blue channel). Stained cells were examined by confocal fluorescence microscopy. Merging of the images in the red, green, and blue channels generated the third image on each row; yellow indicates overlapping localization of the green and red channels. In B, arrows indicate colocalization at ER exit sites. In C, the arrow indicates colocalization of the tip of a tubule containing GM130 with an ER exit site. Bar, 10 μm.

We further investigated the apparent differences between our results using 30 μM H-89 in NRK cells and those obtained by Lee and Linstedt using 50 μM H-89 in HeLa cells [34]. H-89 was first presented as a potent and selective inhibitor of PKA [48]. However, *in vitro* studies have shown that H-89 can also inhibit other serine-threonine protein kinases, like PKCα, although with less potency [49]. For example, 10 μM H-89 almost completely inhibits PKA activity *in vitro*, with a potency almost one order of magnitude higher than for PKCα [49]. This suggests that the different effects on retrograde tubulation could be due to dose dependency or to cell context. To distinguish between these two possibilities we performed dose-response experiments in NRK and HeLa cells. In NRK cells treated with BFA and 10 μM H-89 for 60 min, seemingly no tubules were accumulated, but instead only occurred redistribution of Golgi matrix proteins to ER exit sites (S6B Fig, S8A Fig, S9C Fig, S10A Fig and S11C Fig) or redistribution of *cis*-medial, Golgi resident proteins to the ER (data not shown). In spite of this, we noticed a delay in Golgi to ER transport (S6A Fig), and contacts between the tip of the tubules and ER exit sites were more frequently observed (Fig 4C, S12C Fig and data not shown). Co-incubation of BFA with 50 μM H-89 produced an accumulation of tubules similar to what was observed with 30 μM H-89 (in number per cell and size; S6E and S6F Fig, S8C Fig and S9E Fig), but with lower frequency (in ~20% of cells). Other differences were observed: GM130, GRASP65, p115 and GRASP55 always co-localized in the tubules (S6E and S6F Fig, S10C Fig, S11E Fig and data not shown), but giantin often (~10% of cells containing tubules) was found in tubules or branching tubules non-colocalizing with other Golgi matrix proteins (S8C Fig and S9E Fig). However, in NRK cells treated with myr-PKI-A we never observed giantin in tubules not co-localizing with other Golgi matrix proteins (Fig 2C, S9F Fig, and data not shown). This result suggests that another protein kinase inhibited by 50 μM H-89 regulates the localization of giantin in tubules. Interestingly, in contrast to the other Golgi matrix proteins, giantin is a transmembrane protein [18], suggesting that an unknown protein kinase could regulate protein segregation within Golgi membranes. We also made another intriguing observation: a high proportion of p115 was present in the cytosol (S10C Fig and S11E Fig), but this redistribution was abolished in cells pre-treated with 6-MB-cAMP (data not shown). Because p115 cycles between the cytosol and Golgi membranes [29], this result suggests that PKA may also regulate this process. However, since incubation with myr-PKI-A did not result in redistribution of p115 to the cytosol (Fig 3C), another explanation is that a different kinase regulates the association of p115 with membranes. During interphase the phosphorylation of p115 prevents its association to Golgi membranes [50], thus the effect of 50 μM H-89 on p115 could be indirect. Alternatively, the effects produced by BFA could trigger a distinct regulation of p115 by phosphorylation. Likewise, it is well established that phosphorylation of Golgi matrix proteins is a distinct feature of the disassembly of the Golgi apparatus during mitosis [51]. Therefore, these results reveal that phosphorylation plays additional roles in the regulation of Golgi matrix proteins during interphase. Finally, higher H-89 concentration of up to 100 μM showed no additional features, but a decrease in the percentage of cells with accumulated tubules (~5% of cells), and a (reversible) rounding off of cells (data not shown). In contrast, the accumulation of tubules in HeLa cells containing both Golgi matrix proteins and transmembrane proteins was only observed when treated with BFA and H-89 at a lower concentration of 10–30 μM (data not shown), suggesting that 50 μM H-89 in HeLa cells could inhibit the initiation of tubule formation.

**Fig 5:**
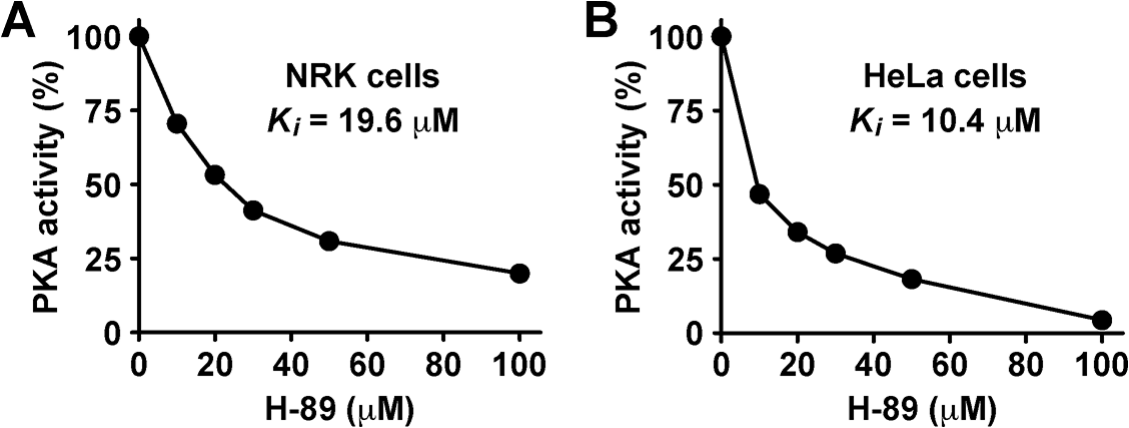
PKA activity in NRK cells is less sensitive to H-89 than in HeLa cells. NRK cells (A) or HeLa cells (B) were left untreated or treated with H-89 at the indicated concentrations for 60 min. Cell extracts were prepared to assess PKA activity using a non-radioactive protein kinase assay kit (Calbiochem), according to the manufacturer's instructions. PKA activity at each concentration of H-89 represents the percentage compared to the activity in untreated cells. N = 3.

Because ATP concentration varies in different cells [41], differing levels of inhibition of PKA activity could explain the variation observed in NRK cells in dose-response experiments. Indeed, we found a reduction of PKA activity to ~71% with 10 μM H-89, to ~41–31% with 30–50 μM, and to ~20% with 100 μM (Fig 5A). Therefore, the differences between NRK and HeLa cells are likely due in part to differential sensitivity to PKA inhibition. In agreement with this possibility, we found that treatment with 50 μM H-89 in HeLa cells resulted in the inhibition of PKA activity to ~18% (Fig 5B). We also performed dose-response experiments in other human cell lines, such as MCF-7 and MDA-MB-231, and found accumulation of tubules at concentrations that ranged between 30 and 50 μM (data not shown). Because H-89 is able to inhibit *in vitro* other protein kinases [49], we cannot discard that 50 μM H-89 is inhibiting additional protein kinases, like the aforementioned PKCα or, as it has been suggested by Lee and Linstedt [34], an unknown serine-threonine protein kinase. Importantly, in NRK cells, no accumulation of tubules, and little to no PKA activity inhibition, was observed in the presence of general PKC inhibitors, like 100 nM calphostin C, 320 nM chelerythrine or 200 μM myr-PKI-C (data not shown). Taken together, H-89 sensitivity seems to explain, at least in part, the differences observed between NRK and HeLa cells. Because the observations were highly reproducible, in the following experiments we used 100 μM myr-PKI-A or 30 μM H-89.

### Accumulated Tubules are Highly Dynamic

**Fig 6:**
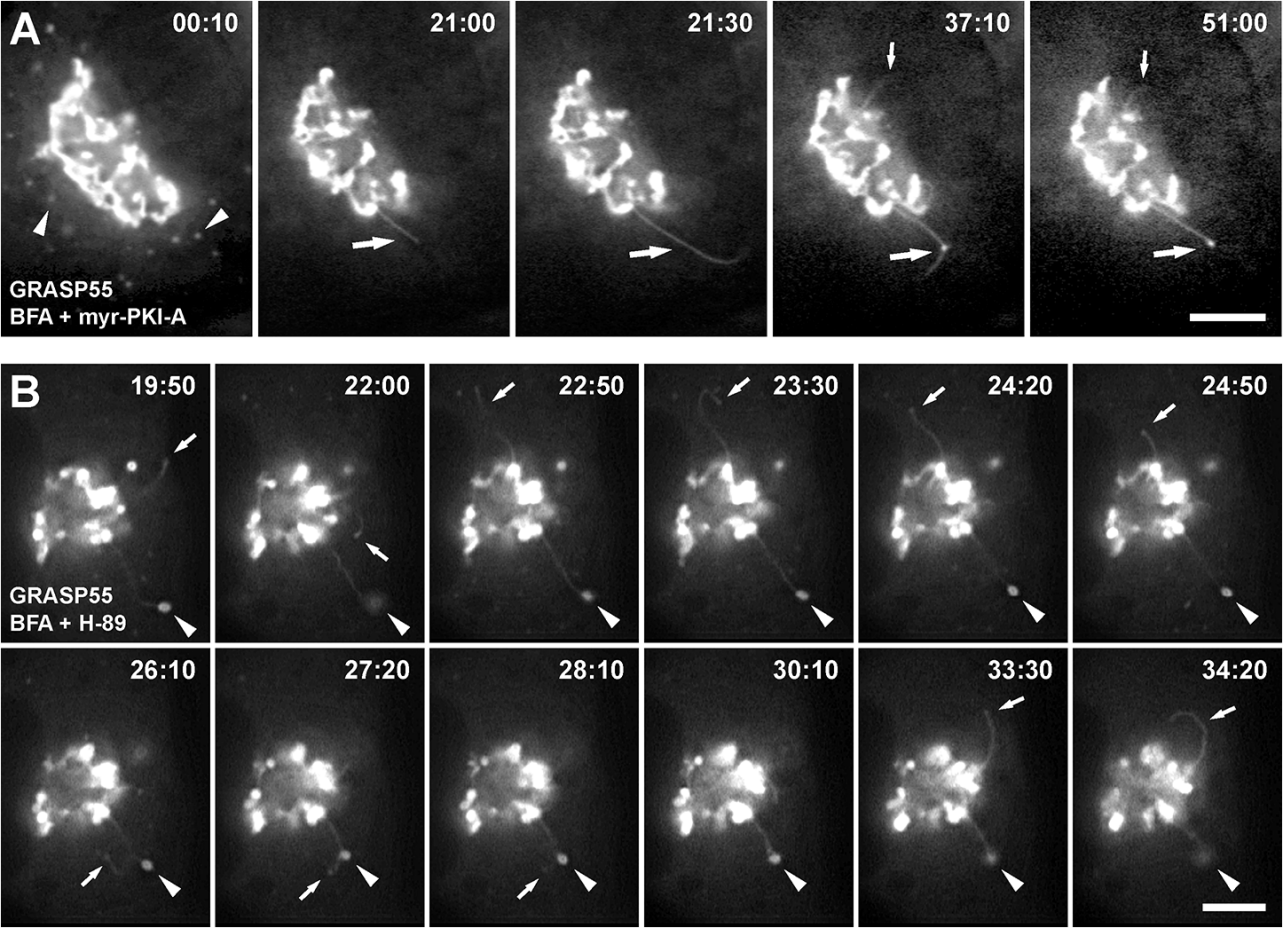
Dynamic behavior of GFP-GRASP55 in accumulated retrograde tubules. NRK cells transiently expressing GFP-GRASP55 were held in a microscope stage at 37°C, and were treated with 5 μg/ml BFA in conjunction with 100 μM myr-PKI-A (A; n = 10) or 30 μM H-89 (B; n = 15). Live cells were examined by fluorescence microscopy, and the time after initiation of each treatment is shown in the upper right corner of each panel in minutes:seconds. In A, arrowheads indicate localization of GFP-GRASP55 at ER exit sites that during the treatment seems to vanish; large arrows indicate the elongation of a tubule (21:00–21:30) that retracts and dilates at a central portion (37:10), and the dilated part remains steady until the end of imaging (51:00); and small arrows indicate a small tubule that elongates at a later time (37:10). In B, small arrows indicate elongation of tubules at different times (19:50, 22:00, 22:50, 26:10, and 33:30), the curving and retraction of a tubule (23:30 to 24:50), the elongation and retraction of a tubule from the central portion of an accumulated tubule (26:10 to 28:10), and the elongation, curving, and retraction of another tubule (33:30 to 34:20); and arrowheads indicate the dilated tip of an accumulated tubule. Bars, 5 μm.

**Fig 7:**
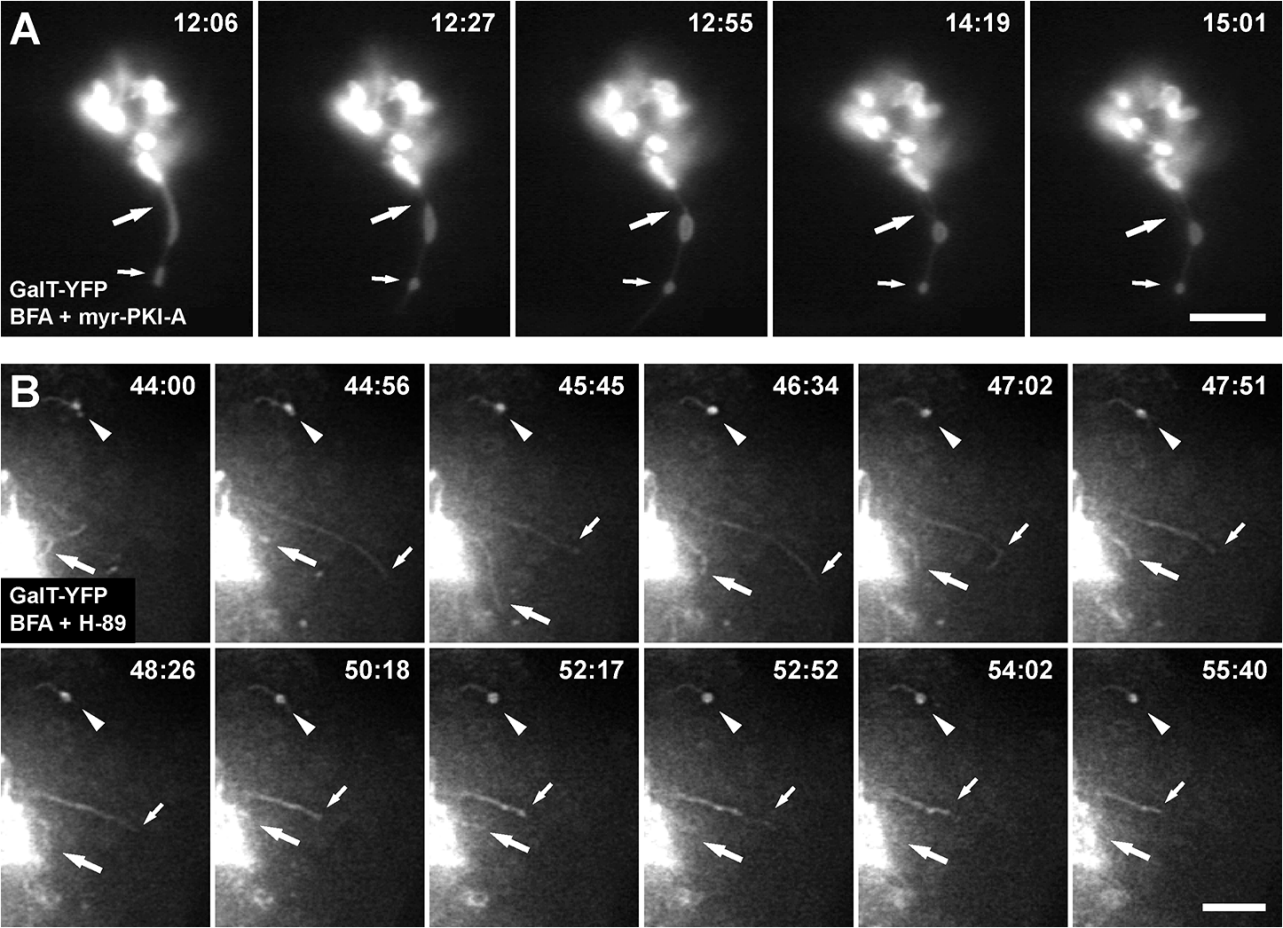
Dynamic behavior of GalT-YFP in accumulated retrograde tubules. NRK cells transiently expressing GalT-YFP were held in a microscope stage at 37°C, and were treated with 5 μg/ml BFA in conjunction with 100 μM myr-PKI-A (A; n = 10) or 30 μM H-89 (B; n = 15). Live cells were examined by fluorescence microscopy, and the time after initiation of each treatment is shown in the upper right corner of each panel in minutes:seconds. In A, large arrows indicate the dwindling of an initial thick tubule that leaves a swollen central portion; and small arrows indicate the formation of a dilated tip. In B, arrowheads indicate the dilated tip of an accumulated tubule; large arrows indicate a tubule that elongates and retracts; and small arrows indicate the elongation and retraction of another tubule. Bars, 5 μm.

The differences observed in size, number and shape of accumulated tubules suggested a highly dynamic process. Thus, to study the dynamics of accumulated tubules, we analyzed Golgi-matrix proteins and Golgi-transmembrane proteins tagged with GFP or its spectral variants. We performed live-cell imaging in NRK cells during treatment with 5 μg/ml BFA alone or co-incubated with 100 μM myr-PKI-A or 30 μM H-89. As reported previously [17], with BFA treatment, retrograde tubules containing GFP-GRASP55 collapsed at or near ER exit sites (S1 Video). In contrast, and in agreement with the immunofluorescence of endogenous GRASP55 (S6 Fig), co-incubation of BFA with myr-PKI-A (Fig 6A, S2 Video and S3 Video), or with H-89 (Fig 6B, S4 Video and S5 Video), showed no redistribution of GFP-GRASP55. Instead, GFP-GRASP55 was associated with accumulated tubules throughout the treatment (up to 2 h). The first noticeable effect was the gradual disappearance of GFP-GRASP55 from peripheral puncta, suggesting that retrograde transport is promptly inhibited while anterograde transport is not affected, at least at the beginning of the treatment (Fig 6A, arrowheads at 00:10 min). Similar behavior was observed in cells expressing GFP-GRASP65 (n = 10), YFP-GM130 (n = 10) or p115-YFP (n = 15) (data not shown), and in cells expressing the construct GalT-YFP (Fig 7A, n = 10; Fig 7B, n = 15, S7 Video and S8 Video).

Similar to fixed cells, the number of accumulated tubules varied from 1 (Figs 6A and 7A) to several, with the majority of cells having 3–4 tubules (Figs 6B and 7B). Tubules were highly dynamic, showing numerous rounds of extension from and retraction to the Golgi, to finally stay steady (Fig 6A and S2 Video). Tubules also bend while they extend, and retract following the same path, consistent with being associated with a cytoskeleton track (Fig 6B, arrows from 22:50 to 24:50 min, and S4 Video). Other tubules emerge and retract from central sections of apparently steady tubules, i.e. tubule branching (Fig 6B, arrows from 26:10 to 28:10 min, and S4 Video). Interestingly, the dynamics of tubule branching seems different compared to that in cells treated only with BFA. While extensive tubulation in cells treated with BFA seems to be the result of a stable building up of tubule branching (S1 Video), retraction of both tubules and branched tubules in cells co-incubated with BFA and PKA inhibitors seems to account for the overall lesser extent in tubulation. Although several attempts have been made to understand membrane tubulation (e.g., [52–54]), the membrane remodeling that facilitate tubule branching is poorly understood, in particular the tubulation that occurs at the Golgi-ER interface. Thus, our observations reveal a mechanism in which protein phosphorylation seems to regulate the interaction of Golgi-to-ER retrograde tubules with microtubules.

Tubules extend and retract independently from each other (Figs 6B and 7B, S4 Video and S8 Video), suggesting that the initiation of tubulation is a stochastic rather than a concerted process. Importantly, pre-incubation with 6-MB-cAMP abolished the distinct dynamic behavior of tubules observed with the inhibitors, resulting in redistribution of Golgi proteins in a manner that was indistinguishable from that of cells treated with BFA only (data not shown). The different morphological aspects were not due to the use of different inhibitors: with each inhibitor we observed elements that were not always present in all cells, at least during the time of imaging, such as tubule branching. It is not clear whether the occurrence of these elements is due to the inhibition of PKA or of other kinases. However, the fact that we observed these additional elements in cells treated with myr-PKI-A, as well as with all the other inhibitors, suggests that PKA is involved.

Like in fixed cells, the tips of some tubules were dilated. This was observed primarily in steady tubules (Fig 6A and 6B, Fig 7A and 7B, S2 Video, S4 Video, S7 Video and S8 Video). Another feature also initially observed in fixed cells was a distension or swelling in the central section of tubules, but experiments in live cells allowed the tracing of the appearance of these structures. In some cases dilation occurred after tubule elongation, presumably by accumulation of material at a certain spot in the tubule. Very often thereafter tubules retracted to- and re-extended from this point, following either the same track (data not shown), or bending following another track, to finally end as a dilated tubule tip (Fig 6A, large arrows from 37:10 to 51:00 min, and S2 Video, S3 Video, S4 Video, S7 Video and S8 Video). In other cases dilation was the result of the dwindling of an initially thicker tubule leaving a swollen portion (Fig 7A and S7 Video), very similar to what we observed in fixed cells (Fig 1D and 1E). Formation of some dilated tubule tips was also preceded by these phenomena (Fig 7A, small arrows, and S7 Video). Together, the behavior of tubules over time suggests that upon reaching a distal point, marked with dilated tips, a final step of transfer of its content (i.e., fusion) is actively blocked. Also, the appearance and disappearance of tubules over time suggests that the number of cells with tubules in fixed cells may have been underestimated.

Finally, NRK cells transiently transfected with CFP-, GFP-, or YFP-tagged Golgi matrix proteins, as well as NRK cells stably expressing GFP-GRASP55, or NRK cells transiently expressing GalT-YFP, were treated for 60 min with BFA and myr-PKI-A or BFA and H-89, fixed and immunolabeled with antibodies against the respective endogenous protein, or antibodies against Man-II, GalNAc-transferase 1 or GalNAc-transferase 2. The tubules containing endogenous Golgi proteins were indistinguishable from those containing the fluorescently tagged proteins (data not shown). Therefore, our conclusion is that the behavior observed in live cells represents true phenomena and not artifacts due to overexpression of exogenous proteins. Altogether, these data suggest that PKA activity is necessary for normal Golgi-to-ER tubular transport.

### PKA activity is necessary for fission of Golgi-to-ER retrograde tubules

**Fig 8:**
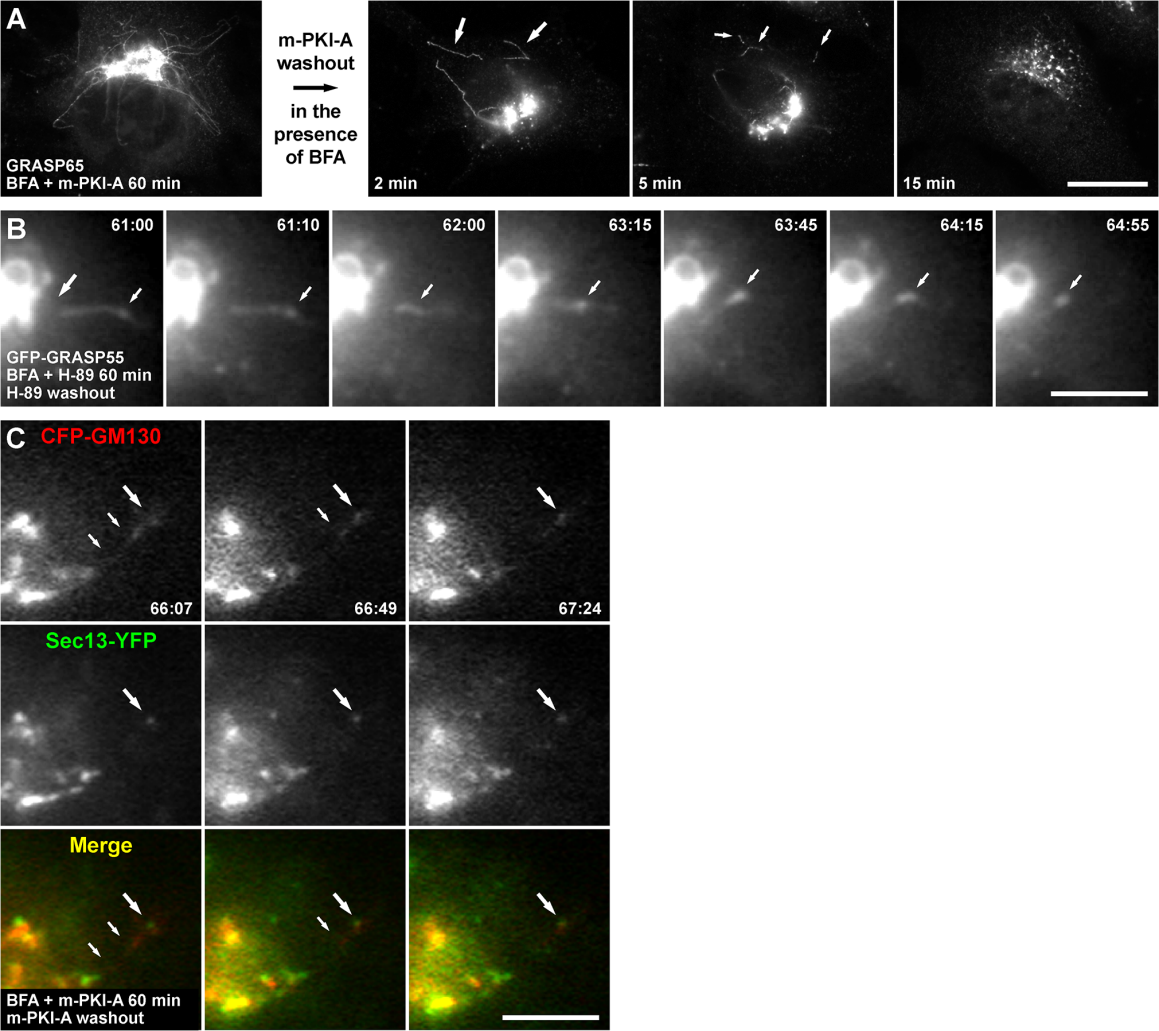
PKA activity is necessary for the fission of accumulated tubules. (A) NRK cells were treated with 5 μg/ml BFA in conjunction with 100 μM myr-PKI-A (m-PKI-A). After 60 min, myr-PKI-A was washed out in the presence of BFA, and cells were fixed at the times indicated. Cells were permeabilized and immunolabeled with rabbit polyclonal antibody to GRASP65, followed by Alexa-488-conjugated donkey anti-rabbit IgG. Stained cells were examined by fluorescence microscopy. Large arrows indicate large cut tubules. Small arrows indicate small cut tubules. Bar, 10 μm. (B) NRK cells transiently expressing GFP-GRASP55 were held in a microscope stage at 37°C. Cells were treated with 5 μg/ml BFA in conjunction with 30 μM H-89, and H-89 was washed out in the presence of BFA (n = 7). The large arrow indicates the site where an accumulated tubule is cut; small arrows indicate a punctum where the cut tubule collapses. Bar, 5 μm. (C) NRK cells transiently co-expressing CFP-GM130 and Sec13-YFP were held in a microscope stage at 37°C. Cells were treated, and myr-PKI-A was washed out as in A (n = 5). Arrows in the upper panels indicate the collapsing of a cut tubule into an ER exit site indicated by a large arrow. Bar, 5 μm. In B and C, live cells were examined by fluorescence microscopy, and the time after initiation of the treatment is shown in the upper or lower right corner of each panel, respectively, in minutes:seconds.

Tubule accumulation was completely reversible, i.e., after 60 min of co-incubation with BFA and myr-PKI-A, followed by washing-out myr-PKI-A in the continuous presence of BFA for 60 more min produced a further redistribution of both Golgi resident transmembrane proteins and Golgi matrix proteins to the ER or ER exit sites, respectively (data not shown). Similar results were obtained with H-89 (data not shown). This suggests that after tubule formation at least two limiting steps are reached: tubule fission from the perinuclear region and tubule fusion with a target membrane. To distinguish which step is being affected by PKA inhibitors two sets of experiments were designed. In the first experiment, NRK cells were co-incubated with BFA and myr-PKI-A for 60 min, then myr-PKI-A was withdrawn in the continuous presence of BFA, followed by fixation at early time points before immunofluorescence with anti-GRASP65. After 2 min of myr-PKI-A withdrawal, we observed cut tubules (Fig 8A), strongly suggesting that PKA activity is necessary for the fission of tubular transport intermediates. These cut tubules were ~5–10 μm in length, and were apparent in ~7% of fixed cells. After 5 min of myr-PKI-A withdrawal, shorter cut tubules of ~1–5 μm (with the majority being 1–2 μm) were present, and were apparent also in ~7% of cells (Fig 8A). Confocal images showed these were truly cut tubules (data not shown). Concomitantly with tubule disappearance, GRASP65 redistributed to ER exit sites, and this redistribution increased over time, to be apparently complete after 15 min of myr-PKI-A withdrawal (Fig 8A, and data not shown). Cut tubules were also observed containing Man-II with a posterior redistribution toward the ER upon myr-PKI-A withdrawal (data not shown). Similar results were obtained using H-89, however the kinetics of cut tubule generation seemed to be faster (S13A Fig), due to different kinetics of inhibitor dissociation or different cell permeability to the inhibitor. The same experiment was done in NRK cells expressing GFP-GRASP55 (Fig 8B and S9 Video), or co-expressing CFP-GM130 and Sec13-YFP (Fig 8C). Fig 8B shows an example of a cell treated with H-89, right in the moment when the tubule was cut from the Golgi (large arrow at 61:00 min, and S9 Video). The cut tubule rapidly decreased in length during the first minute of H-89 withdrawal, to finally collapse in a spot that remained stable afterward (Fig 8B, small arrows from 63:45 to 64:55). The spots where tubules collapsed seemed to be in close proximity to ER exit sites, as shown by colocalization of CFP-GM130 and Sec13-YFP (Fig 8C). These data support the conclusion that PKA activity is necessary for fission of Golgi-to-ER tubules.

### PKA activity is necessary for fusion of Golgi-to-ER retrograde tubules

**Fig 9:**
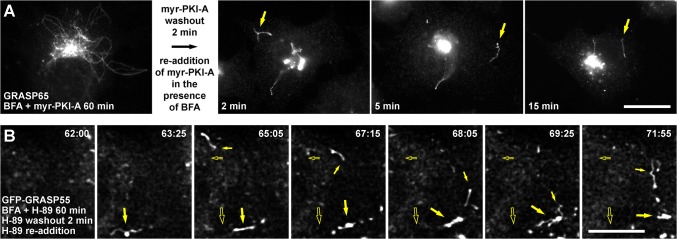
PKA activity is necessary for the fusion of cut accumulated tubules. (A) NRK cells were treated with 5 μg/ml BFA in conjunction with 100 μM myr-PKI-A. After 60 min, myr-PKI-A was washed out for 2 min in the presence of BFA, and 100 μM myr-PKI-A was re-added in the presence of BFA. Cells were fixed at the times indicated, permeabilized and immunolabeled with rabbit polyclonal antibody to GRASP65, followed by Alexa-488-conjugated donkey anti-rabbit IgG. Arrows indicate accumulated cut tubules. Bar, 10 μm. (B) NRK cells transiently expressing GFP-GRASP55 were held in a microscope stage at 37°C. Cells were treated with 5 μg/ml BFA in conjunction with 30 μM H-89, and H-89 was washed out for 2 min in the presence of BFA, and 30 μM H-89 was re-added in the presence of BFA (n = 7). Live cells were examined by fluorescence microscopy, and the time after initiation of the treatment is shown in the upper right corner of each panel in minutes:seconds. Large arrows indicate a mobile cut tubule that collapses at a punctum from where re-emerges to continue mobilizing in the cytoplasm; small arrows indicate another mobile cut tubule that behaves in a very protean fashion. Empty arrows indicate the initial position of cut tubules. Bar, 5 μm.

To determine if tubule fusion is also inhibited, we added myr-PKI-A in two rounds. First, NRK cells were treated with BFA and myr-PKI-A for 60 min, treatment that results in the accumulation of tubules (Fig 9A). To allow tubules to get cut, myr-PKI-A was washed out for 2 min in the continuous presence of BFA. Immediately after, myr-PKI-A was re-added and co-incubated with BFA for different times, followed by fixation and immunofluorescence with anti-GRASP65. In this condition, cut tubules of ~5 μm accumulated, observed even after 15 min of myr-PKI-A re-addition (Fig 9A). We obtained a similar result using H-89 (S13B Fig), as well as using 4-cyano-3-methylisoquinoline or *R*_p_-cAMPS (data not shown). However, with these inhibitors we observed the accumulation of longer cut tubules (~5–12 μm), presumably due to different kinetics of inhibition that was influenced by different cell permeability. Prolonged co-incubation for 60 min after myr-PKI-A or H-89 re-addition still showed cut tubules, but with reduced size (~2–7 μm; data not shown). Re-addition of myr-PKI-A or H-89 after 10 min of initial withdrawal showed little or no accumulation of cut tubules, suggesting that a short time is necessary to fuse with- or collapse close to- ER exit sites. When this experiment was monitored in live NRK cells stably expressing GFP-GRASP55 (n = 5; Fig 9B, and S10 Video), or GalT-YFP (n = 5; data not shown), we made the striking observation that cut tubules were highly mobile, and behaved in a very protean fashion, both in cells treated with myr-PKI-A (data not shown) or with H-89 (Fig 9B, and S10 Video). For instance, cut tubules advance in any direction, some retract and move in the opposite direction, some stay immobile to later resume movement to any direction, and some seem to collapse in a place, remaining stable there, but later re-emerge to continue the movement (Fig 9B, and S10 Video). These observations suggest that after re-addition of myr-PKI-A or H-89, the fusion of cut tubules with (or close to) ER exit sites is also inhibited. Importantly, adding 6-MB-cAMP when myr-PKI-A or H-89 was re-added abolished accumulation of cut tubules (data not shown). Hence, from all the data presented we conclude that PKA activity is necessary for both fission and fusion of Golgi-to-ER retrograde tubules. Then, what are the targets of PKA? Only a few proteins are known to function in Golgi-to-ER tubulation. Among them are phospholipid remodeling enzymes [55], and components of the CtBP1/BARS-dependent fission machinery [56]. However, PKA can phosphorylate a large number of Golgi proteins [57]. Therefore, further analysis will be needed to dissect the molecular elements regulated by PKA that participate in the fission and fusion processes of this membrane transport pathway.

## Supporting Information

S1 Fig Redistribution of β-COP by BFA and initiation of tubulation are not affected by PKA inhibitors. NRK cells were left untreated (A) or treated with 5 μg/ml BFA in conjunction with either 30 μM H-89 (B) or 100 μM myr-PKI-A (C), for the time indicated. Cells were fixed, permeabilized, immunolabeled with rabbit antibody to GM130 and mouse monoclonal antibody to β-COP, followed by Alexa-594-conjugated donkey anti-rabbit IgG (red channel), and Alexa-488-conjugated donkey anti-mouse IgG (green channel), respectively. Nuclei were stained with DAPI (blue channel). Stained cells were examined by fluorescence microscopy. Merging of the images in the red, green, and blue channels generated the third image on each row; yellow indicates overlapping localization of the green and red channels. Arrows indicate absence of β-COP in elongating tubules. Bar, 10 μm.
(TIF)

S2 Fig PKA inhibitors do not affect initiation of tubulation of tubules containing GM130 and GRASP65. NRK cells were left untreated (A) or treated with 5 μg/ml BFA in conjunction with either 30 μM H-89 (B) or 100 μM myr-PKI-A (C), for the time indicated. Cells were fixed, permeabilized, immunolabeled with mouse monoclonal antibody to GM130 and rabbit antibody to GRASP65, followed by Alexa-594-conjugated donkey anti-mouse IgG (red channel), and Alexa-488-conjugated donkey anti-rabbit IgG (green channel), respectively. Nuclei were stained with DAPI (blue channel). Stained cells were examined by fluorescence microscopy. Merging of the images in the red, green, and blue channels generated the third image on each row; yellow indicates overlapping localization of the green and red channels. Arrows indicate colocalization in elongating tubules. Bar, 10 μm.
(TIF)

S3 Fig PKA inhibitors do not affect initiation of tubulation of tubules containing GRASP55 and GRASP65. NRK cells were left untreated (A) or treated with 5 μg/ml BFA in conjunction with either 30 μM H-89 (B) or 100 μM myr-PKI-A (C), for the time indicated. Cells were fixed, permeabilized, immunolabeled with mouse monoclonal antibody to GRASP55 and rabbit antibody to GRASP65, followed by Alexa-594-conjugated donkey anti-mouse IgG (red channel), and Alexa-488-conjugated donkey anti-rabbit IgG (green channel), respectively. Nuclei were stained with DAPI (blue channel). Stained cells were examined by fluorescence microscopy. Merging of the images in the red, green, and blue channels generated the third image on each row; yellow indicates overlapping localization of the green and red channels. Arrows indicate colocalization in elongating tubules. Bar, 10 μm.
(TIF)

S4 Fig Branching of accumulated tubules is less pronounced than in tubules of cells treated only with BFA. NRK cells were treated with 5 μg/ml BFA (A), or with BFA in conjunction with either 30 μM H-89 (B) or 100 μM myr-PKI-A (C), for the indicated times. Cells were fixed, permeabilized, immunolabeled with goat polyclonal antibody to GRASP65, followed by Alexa-488-conjugated donkey anti-goat IgG (green channel), and nuclei were stained with DAPI (blue channel). Stained cells were examined by fluorescence microscopy. Bar, 10 μm.
(TIF)

S5 Fig The continuity of Golgi and tubules membranes is maintained upon PKA inhibition during treatment with BFA. NRK cells stably expressing GFP-GRASP55 were held in a microscope stage at 37°C, and treated with 5 μg/ml BFA in conjunction with 30 μM H-89. After 60 min cells were analyzed by laser confocal microscopy acquiring images at various depths indicated in the top right corner of each panel. The last image depicts the projection of the images acquired along the optical axis. Arrows indicate tubules that connect elements of the Golgi apparatus. Bar, 5 μm.
(TIF)

S6 Fig Accumulation of tubules depends on the dose of H-89. NRK cells were treated with 5 μg/ml BFA in conjunction with the indicated concentrations of H-89, and for the time indicated. Cells were fixed, permeabilized, immunolabeled with mouse monoclonal antibody to GRASP55 and goat polyclonal antibody to GRASP65, followed by Alexa-594-conjugated donkey anti-mouse IgG (red channel) and Alexa-488-conjugated donkey anti-goat IgG (green channel), and nuclei were stained with DAPI (blue channel). Stained cells were examined by fluorescence microscopy. Merging of the images in the red, green, and blue channels generated the third image on each row; yellow indicates overlapping localization of the green and red channels. Arrows indicate colocalization at dilated tips. Bar, 10 μm.
(TIF)

S7 Fig Accumulation of tubules is reversible, can result from the treatment with H-89 or myr-PKI-A only, and is prevented by 6-MB-cAMP. NRK cells were treated with 5 μg/ml BFA in conjunction with 30 μM H-89 for 60 min followed by washout of both compounds for 60 min (A), or treated with 30 μM H-89 for 60 min (B), or treated with 5 μg/ml BFA in conjunction with 100 μM myr-PKI-A for 60 min followed by washout of both compounds for 60 min (C), or treated with 100 μM myr-PKI-A for 60 min (D), or pre-incubated 5 min with 200 μM 6-MB-cAMP followed by addition of 5 μg/ml BFA and 30 μM H-89 for 60 min (E). Cells were fixed, permeabilized, immunolabeled with mouse monoclonal antibody to GM130 and rabbit antibody to α-mannosidase II (Man-II), followed by Alexa-594-conjugated donkey anti-mouse IgG (red channel), and Alexa-488-conjugated donkey anti-rabbit IgG (green channel). Nuclei were stained with DAPI (blue channel). Stained cells were examined by confocal fluorescence microscopy. Merging of the images in the red, green, and blue channels generated the third image on each row; yellow indicates overlapping localization of the green and red channels. In B, arrowheads indicate colocalization at accumulated tubules, and arrows indicate colocalization at a swollen portion of a tubule. In E, arrows indicate colocalization at ER exit sites. Bar, 10 μm.
(TIF)

S8 Fig Giantin and GRASP65 accumulate distinctly in retrograde tubules upon different concentrations of H-89. NRK cells were treated with 5 μg/ml BFA in conjunction with H-89 at the indicated concentration (A-C), or pre-incubated 5 min with 200 μM 6-MB-cAMP followed by addition of BFA and 30 μM H-89 (D), for the time indicated. Cells were fixed, permeabilized, immunolabeled with mouse monoclonal antibody to Giantin and rabbit antibody to GRASP65, followed by Alexa-594-conjugated donkey anti-mouse IgG (red channel), and Alexa-488-conjugated donkey anti-rabbit IgG (green channel). Nuclei were stained with DAPI (blue channel). Stained cells were examined by fluorescence microscopy. Merging of the images in the red, green, and blue channels generated the third image on each row; yellow indicates overlapping localization of the green and red channels. In A, arrows indicate colocalization at ER exit sites. In B, arrows indicate colocalization at accumulated tubules. In C, the arrow indicates colocalization in an accumulated tubule; empty arrowheads indicate tubules containing only Giantin; and filled arrowheads indicate tubules containing only GRASP65. Bar, 10 μm.
(TIF)

S9 Fig Different treatments with BFA and PKA inhibitors result in distinct effects on Giantin and GRASP65. NRK cells were left untreated (A) or treated with 5 μg/ml BFA (B), or with BFA in conjunction with either H-89 at the indicated concentration (C-E) or 100 μM myr-PKI-A (F), or pre-incubated 5 min with 200 μM 6-MB-cAMP followed by addition of 5 μg/ml BFA and 30 μM H-89 (G), for the time indicated. Cells were fixed, permeabilized, immunolabeled with mouse monoclonal antibody to Giantin and rabbit antibody to GRASP65, followed by Alexa-594-conjugated donkey anti-mouse IgG (red channel), and Alexa-488-conjugated donkey anti-rabbit IgG (green channel). Nuclei were stained with DAPI (blue channel). Stained cells were examined by fluorescence microscopy. Merging of the images in the red, green, and blue channels generated the third image on each row; yellow indicates overlapping localization of the green and red channels. Bar, 10 μm.
(TIF)

S10 Fig P115 and GM130 accumulate distinctly in retrograde tubules upon different concentrations of H-89. NRK cells were treated with 5 μg/ml BFA in conjunction with H-89 at the indicated concentration (A-C), or pre-incubated 5 min with 200 μM 6-MB-cAMP followed by addition of BFA and 30 μM H-89 (D), for the time indicated. Cells were fixed, permeabilized, immunolabeled with rabbit polyclonal antibody to p115 and mouse monoclonal antibody to GM130, followed by Alexa-594-conjugated donkey anti-rabbit IgG (red channel), and Alexa-488-conjugated donkey anti-mouse IgG (green channel). Nuclei were stained with DAPI (blue channel). Stained cells were examined by fluorescence microscopy. Merging of the images in the red, green, and blue channels generated the third image on each row; yellow indicates overlapping localization of the green and red channels. In A, arrows indicate colocalization at ER exit sites. In B, arrows indicate colocalization at accumulated tubules; and arrowheads indicate localization of p115 at ER exit sites. In C, arrows indicate colocalization in accumulated tubules. Bar, 10 μm.
(TIF)

S11 Fig Different treatments with BFA and PKA inhibitors result in distinct effects on p115 and GM130. NRK cells were left untreated (A) or treated with 5 μg/ml BFA (B), or with BFA in conjunction with either H-89 at the indicated concentration (C-E) or 100 μM myr-PKI-A (F), or pre-incubated 5 min with 200 μM 6-MB-cAMP followed by addition of BFA and 30 μM H-89 (G), for the time indicated. Cells were fixed, permeabilized, immunolabeled with rabbit polyclonal antibody to p115 and mouse monoclonal antibody to GM130, followed by Alexa-594-conjugated donkey anti-rabbit IgG (red channel), and Alexa-488-conjugated donkey anti-mouse IgG (green channel). Nuclei were stained with DAPI (blue channel). Stained cells were examined by fluorescence microscopy. Merging of the images in the red, green, and blue channels generated the third image on each row; yellow indicates overlapping localization of the green and red channels. Bar, 10 μm.
(TIF)

S12 Fig The tip of tubules containing GM130 colocalizes with ER exit sites. NRK cells were left untreated (A) or treated with 5 μg/ml BFA (B), or with BFA in conjunction with 100 μM myr-PKI-A (C), for the time indicated. Cells were fixed, permeabilized, immunolabeled with mouse monoclonal antibody to GM130 and rabbit antibody to Sec23A, followed by Alexa-594-conjugated donkey anti-mouse IgG (red channel), and Alexa-488-conjugated donkey anti-rabbit IgG (green channel). Nuclei were stained with DAPI (blue channel). Stained cells were examined by confocal fluorescence microscopy. Merging of the images in the red, green, and blue channels generated the third image on each row; yellow indicates overlapping localization of the green and red channels. In C, arrows indicate colocalization of tips of tubules or dilated portion of tubules with ER exit sites. Bar, 10 μm.
(TIF)

S13 Fig Fission of accumulated tubules and fusion of cut accumulated tubules is precluded by H-89. NRK cells were treated with 5 μg/ml BFA in conjunction with 30 μM H-89 (A-B). After 60 min, H-89 was either washed out in the presence of BFA, and cells were fixed at the times indicated (A), or H-89 was washed out for 30 sec in the presence of BFA, followed by re-addition of 30 μM H-89 in the presence of BFA, and cells were fixed at the times indicated (B). Cells were permeabilized and immunolabeled with rabbit polyclonal antibody to GRASP65, followed by Alexa-488-conjugated donkey anti-rabbit IgG. Stained cells were examined by fluorescence microscopy. Large arrows indicate large cut tubules. Small arrows indicate small cut tubules. Bar, 10 μm.
(TIF)

S1 Video Redistribution of GFP-GRASP55 in NRK cells treated with BFA. (MOV)

S2 Video Effect on GFP-GRASP55 in NRK cells treated with BFA and myr-PKI-A. (MOV)

S3 Video Accumulation of tubules containing GFP-GRASP55 in NRK cells treated with BFA and myr-PKI-A. (MOV)

S4 Video The accumulation of tubules containing GFP-GRASP55 in NRK cells treated with BFA and H-89 is similar to the accumulation of tubules in cells treated with BFA and myr-PKI-A. (MOV)

S5 Video The effect on GFP-GRASP55 in NRK cells treated with BFA and H-89 is similar to the effect in cells treated with BFA and myr-PKI-A. (MOV)

S6 Video Redistribution of GalT-YFP in NRK cells treated with BFA. (MOV)

S7 Video Effect on GalT-YFP in NRK cells treated with BFA and myr-PKI-A. (MOV)

S8 Video Effect on GalT-YFP in NRK cells treated with BFA and H-89. (MOV)

S9 Video An accumulated tubule containing GFP-GRASP55 is cut upon H-89 withdrawal. (MOV)

S10 Video Dynamic behavior of cut tubules containing GFP-GRASP55 after re-addition of H-89. (MOV)
