# Supplementary figures and images for: Protein Kinase A Activity Is Necessary for Fission and Fusion of Golgi to Endoplasmic Reticulum Retrograde Tubules
Source: PLoS One. 2015 Aug 10;10(8):e0135260. doi: 10.1371/journal.pone.0135260 (PMC4530959; doi:10.1371/journal.pone.0135260)

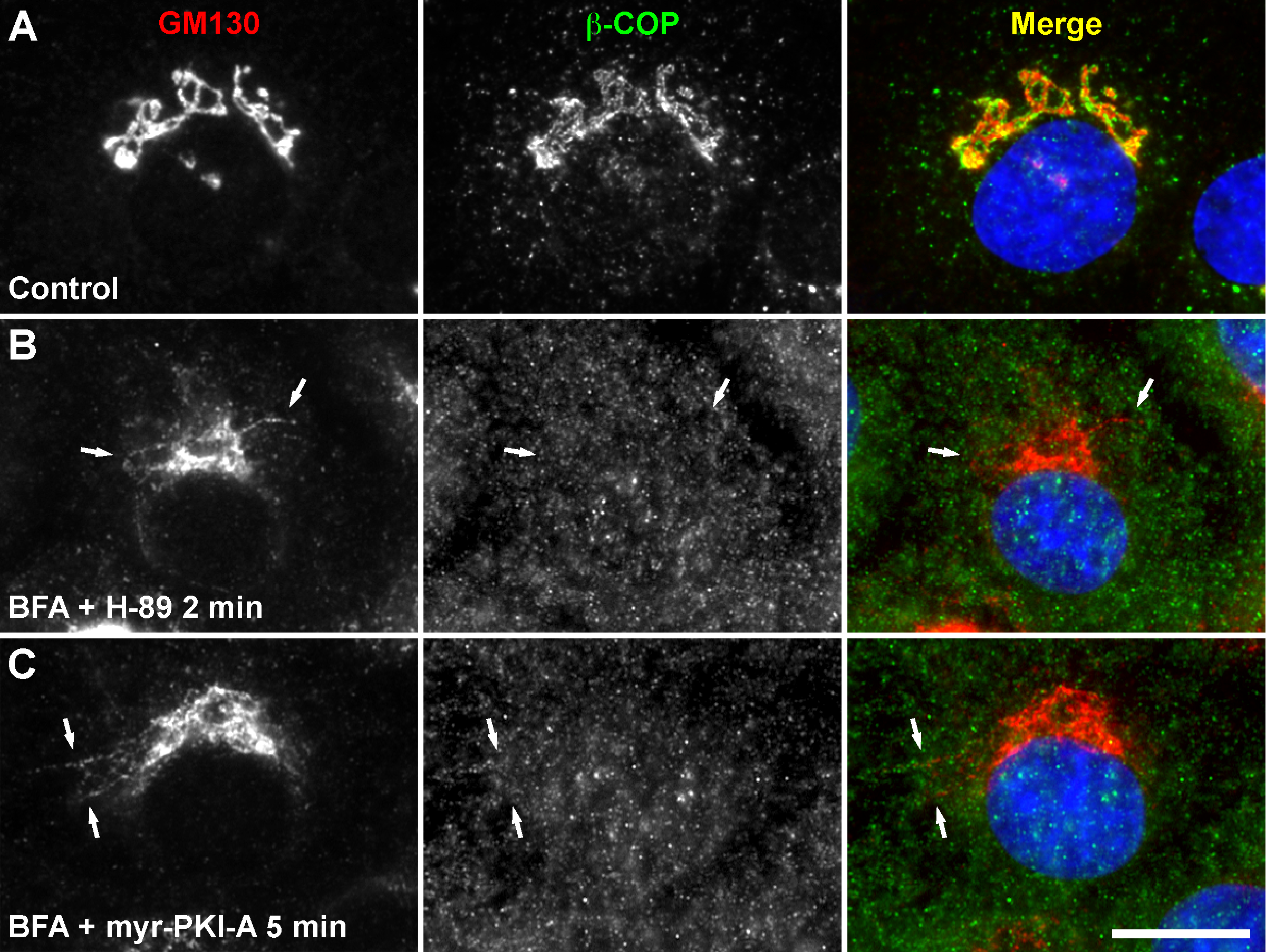

Supplement: S1 Fig — NRK cells were left untreated (A) or treated with 5 μg/ml BFA in conjunction with either 30 μM H-89 (B) or 100 μM myr-PKI-A (C), for the time indicated. Cells were fixed, permeabilized, immunolabeled with rabbit antibody to GM130 and mouse monoclonal antibody to β-COP, followed by Alexa-594-conjugated donkey anti-rabbit IgG (red channel), and Alexa-488-conjugated donkey anti-mouse IgG (green channel), respectively. Nuclei were stained with DAPI (blue channel). Stained cells were examined by fluorescence microscopy. Merging of the images in the red, green, and blue channels generated the third image on each row; yellow indicates overlapping localization of the green and red channels. Arrows indicate absence of β-COP in elongating tubules. Bar, 10 μm. (TIF) [file pone.0135260.s001.tif]

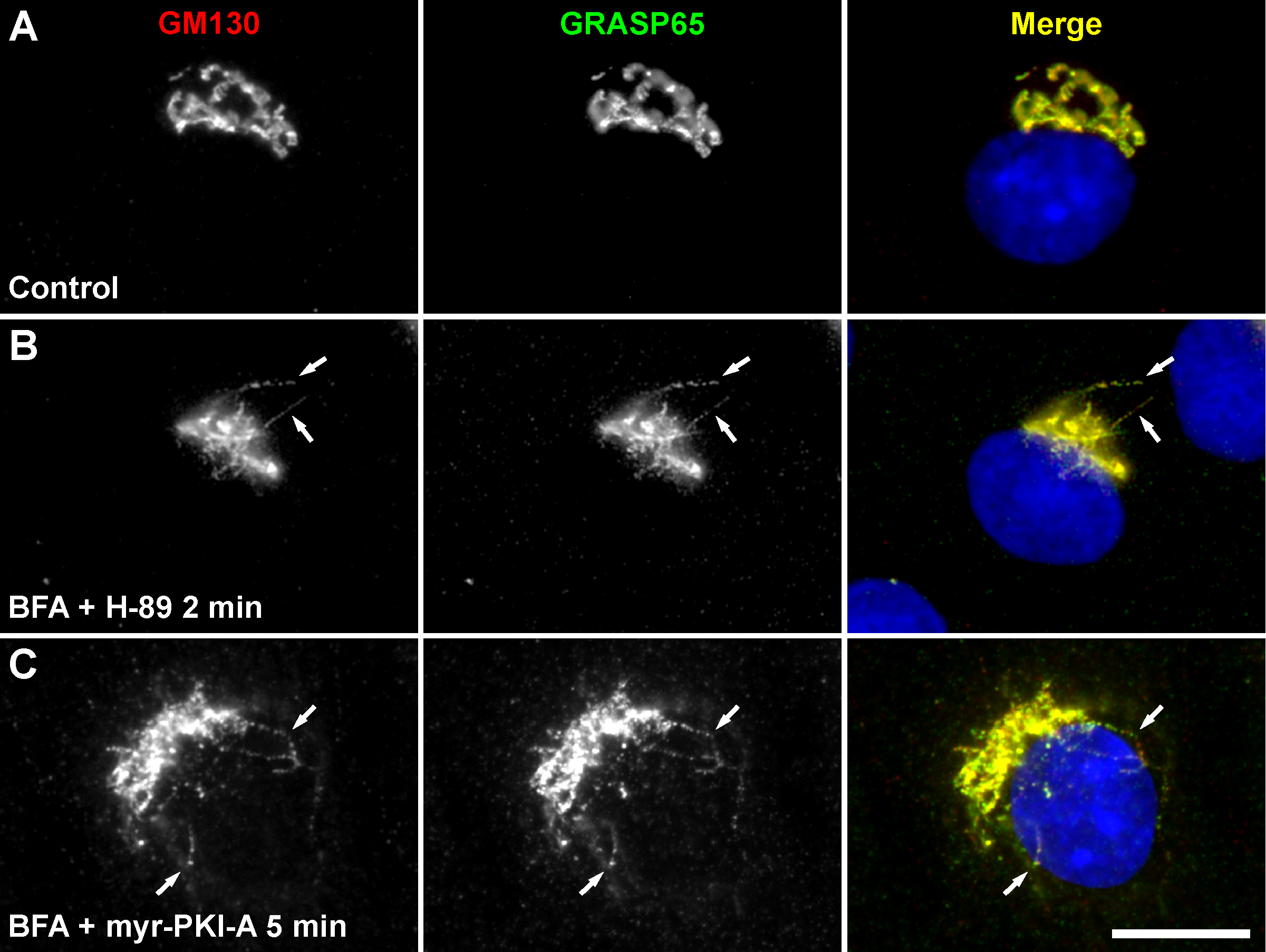

Supplement: S2 Fig — NRK cells were left untreated (A) or treated with 5 μg/ml BFA in conjunction with either 30 μM H-89 (B) or 100 μM myr-PKI-A (C), for the time indicated. Cells were fixed, permeabilized, immunolabeled with mouse monoclonal antibody to GM130 and rabbit antibody to GRASP65, followed by Alexa-594-conjugated donkey anti-mouse IgG (red channel), and Alexa-488-conjugated donkey anti-rabbit IgG (green channel), respectively. Nuclei were stained with DAPI (blue channel). Stained cells were examined by fluorescence microscopy. Merging of the images in the red, green, and blue channels generated the third image on each row; yellow indicates overlapping localization of the green and red channels. Arrows indicate colocalization in elongating tubules. Bar, 10 μm. (TIF) [file pone.0135260.s002.tif]

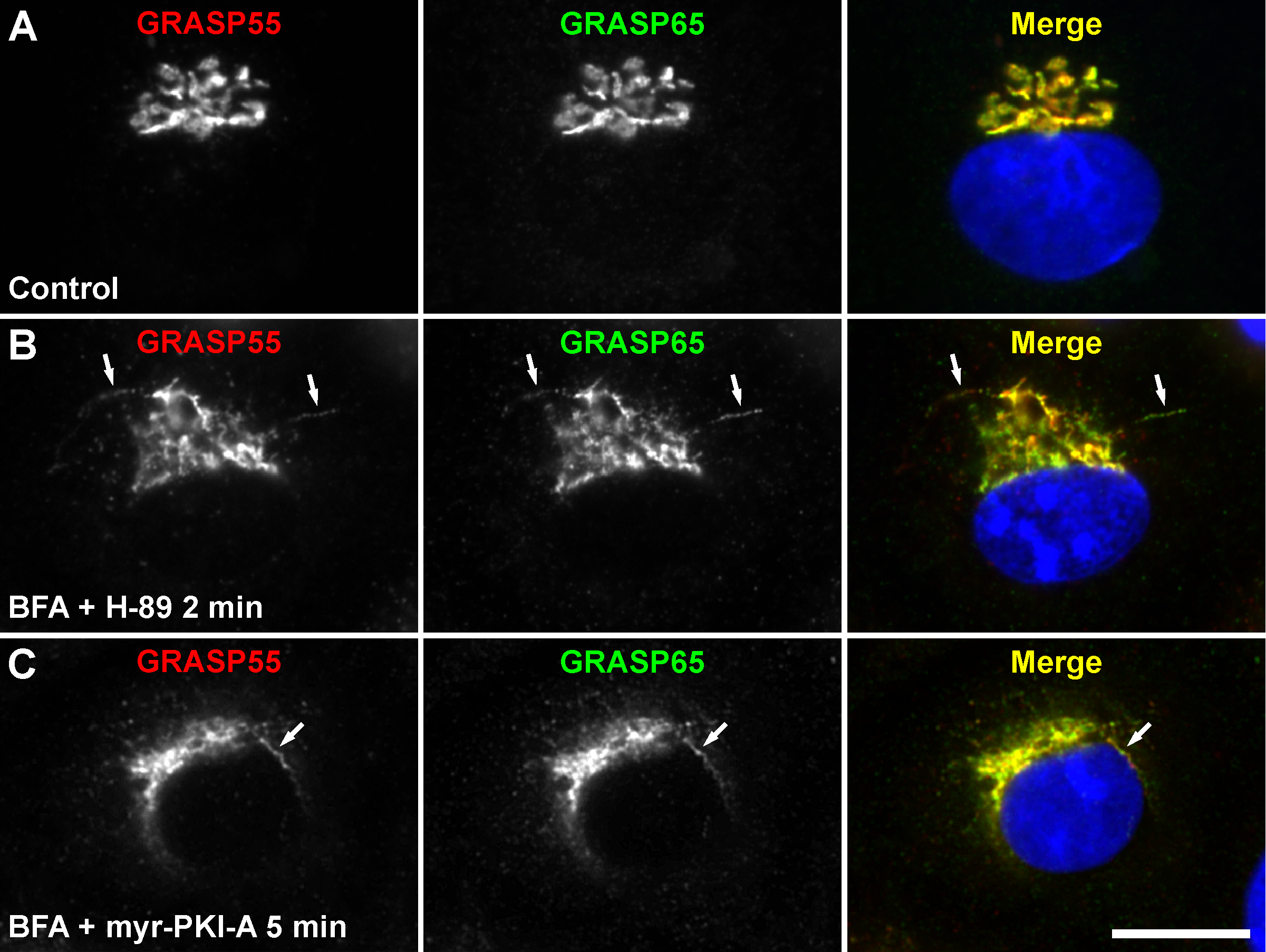

Supplement: S3 Fig — NRK cells were left untreated (A) or treated with 5 μg/ml BFA in conjunction with either 30 μM H-89 (B) or 100 μM myr-PKI-A (C), for the time indicated. Cells were fixed, permeabilized, immunolabeled with mouse monoclonal antibody to GRASP55 and rabbit antibody to GRASP65, followed by Alexa-594-conjugated donkey anti-mouse IgG (red channel), and Alexa-488-conjugated donkey anti-rabbit IgG (green channel), respectively. Nuclei were stained with DAPI (blue channel). Stained cells were examined by fluorescence microscopy. Merging of the images in the red, green, and blue channels generated the third image on each row; yellow indicates overlapping localization of the green and red channels. Arrows indicate colocalization in elongating tubules. Bar, 10 μm. (TIF) [file pone.0135260.s003.tif]

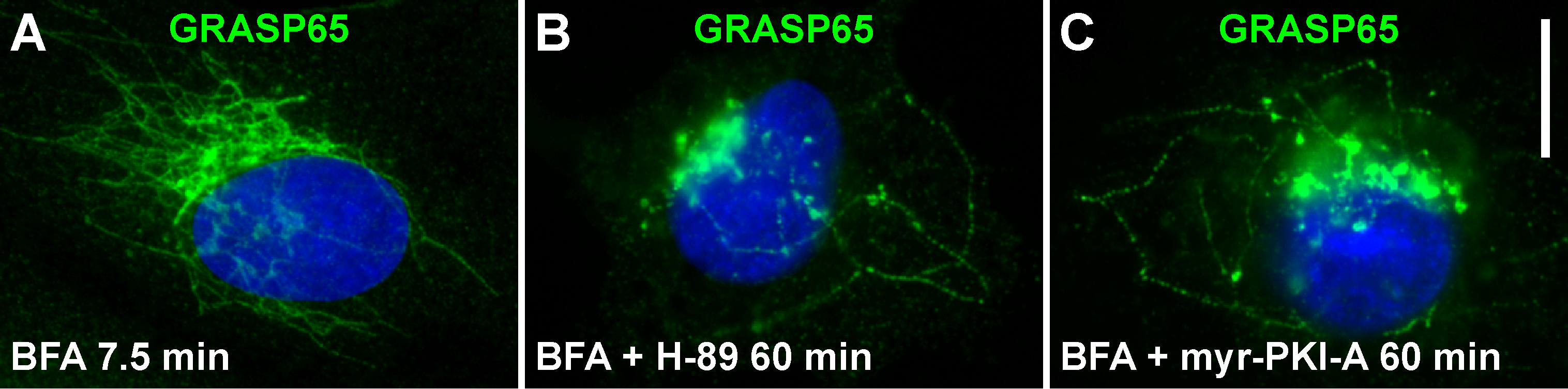

Supplement: S4 Fig — NRK cells were treated with 5 μg/ml BFA (A), or with BFA in conjunction with either 30 μM H-89 (B) or 100 μM myr-PKI-A (C), for the indicated times. Cells were fixed, permeabilized, immunolabeled with goat polyclonal antibody to GRASP65, followed by Alexa-488-conjugated donkey anti-goat IgG (green channel), and nuclei were stained with DAPI (blue channel). Stained cells were examined by fluorescence microscopy. Bar, 10 μm. (TIF) [file pone.0135260.s004.tif]

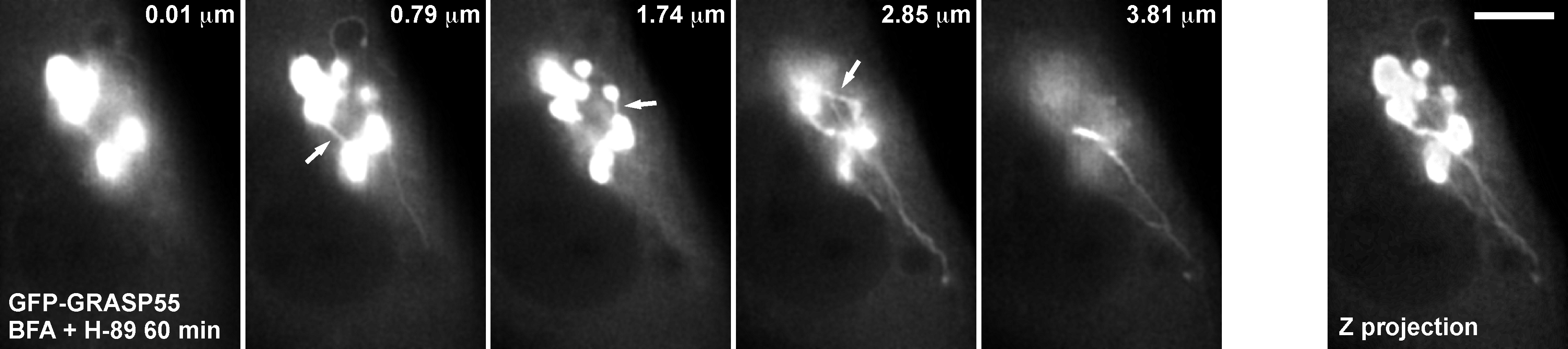

Supplement: S5 Fig — NRK cells stably expressing GFP-GRASP55 were held in a microscope stage at 37°C, and treated with 5 μg/ml BFA in conjunction with 30 μM H-89. After 60 min cells were analyzed by laser confocal microscopy acquiring images at various depths indicated in the top right corner of each panel. The last image depicts the projection of the images acquired along the optical axis. Arrows indicate tubules that connect elements of the Golgi apparatus. Bar, 5 μm. (TIF) [file pone.0135260.s005.tif]

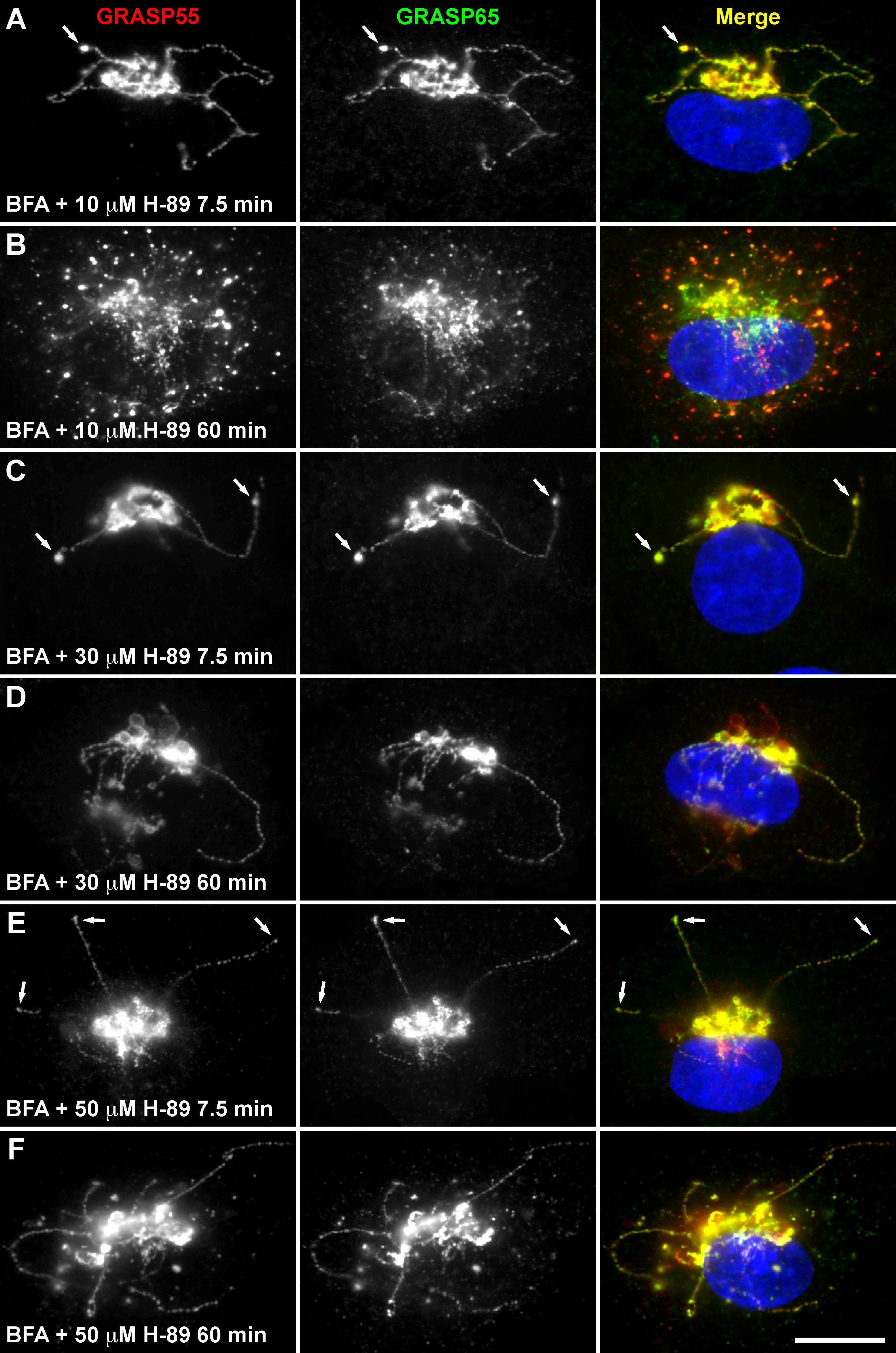

Supplement: S6 Fig — NRK cells were treated with 5 μg/ml BFA in conjunction with the indicated concentrations of H-89, and for the time indicated. Cells were fixed, permeabilized, immunolabeled with mouse monoclonal antibody to GRASP55 and goat polyclonal antibody to GRASP65, followed by Alexa-594-conjugated donkey anti-mouse IgG (red channel) and Alexa-488-conjugated donkey anti-goat IgG (green channel), and nuclei were stained with DAPI (blue channel). Stained cells were examined by fluorescence microscopy. Merging of the images in the red, green, and blue channels generated the third image on each row; yellow indicates overlapping localization of the green and red channels. Arrows indicate colocalization at dilated tips. Bar, 10 μm. (TIF) [file pone.0135260.s006.tif]

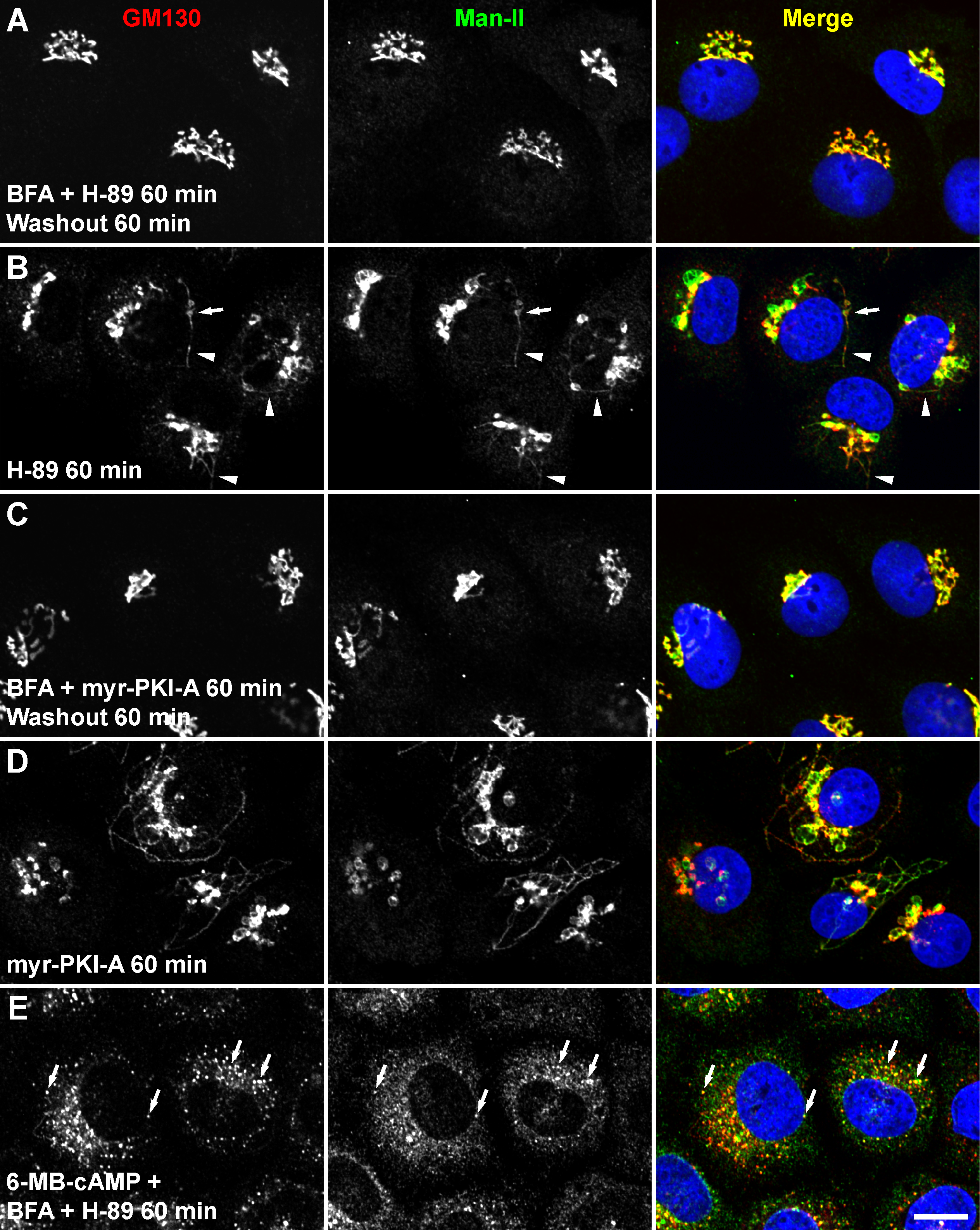

Supplement: S7 Fig — NRK cells were treated with 5 μg/ml BFA in conjunction with 30 μM H-89 for 60 min followed by washout of both compounds for 60 min (A), or treated with 30 μM H-89 for 60 min (B), or treated with 5 μg/ml BFA in conjunction with 100 μM myr-PKI-A for 60 min followed by washout of both compounds for 60 min (C), or treated with 100 μM myr-PKI-A for 60 min (D), or pre-incubated 5 min with 200 μM 6-MB-cAMP followed by addition of 5 μg/ml BFA and 30 μM H-89 for 60 min (E). Cells were fixed, permeabilized, immunolabeled with mouse monoclonal antibody to GM130 and rabbit antibody to α-mannosidase II (Man-II), followed by Alexa-594-conjugated donkey anti-mouse IgG (red channel), and Alexa-488-conjugated donkey anti-rabbit IgG (green channel). Nuclei were stained with DAPI (blue channel). Stained cells were examined by confocal fluorescence microscopy. Merging of the images in the red, green, and blue channels generated the third image on each row; yellow indicates overlapping localization of the green and red channels. In B, arrowheads indicate colocalization at accumulated tubules, and arrows indicate colocalization at a swollen portion of a tubule. In E, arrows indicate colocalization at ER exit sites. Bar, 10 μm. (TIF) [file pone.0135260.s007.tif]

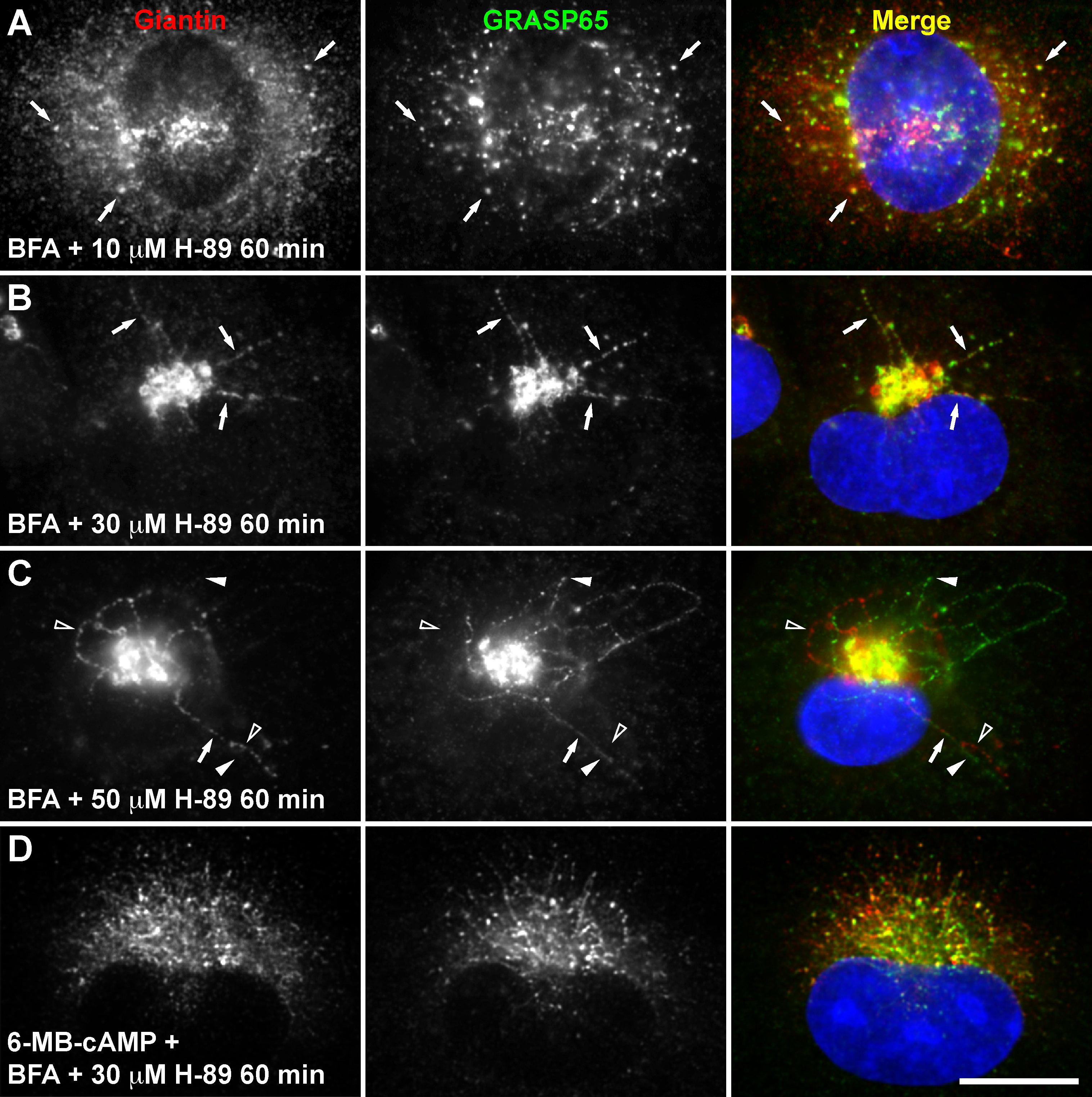

Supplement: S8 Fig — NRK cells were treated with 5 μg/ml BFA in conjunction with H-89 at the indicated concentration (A-C), or pre-incubated 5 min with 200 μM 6-MB-cAMP followed by addition of BFA and 30 μM H-89 (D), for the time indicated. Cells were fixed, permeabilized, immunolabeled with mouse monoclonal antibody to Giantin and rabbit antibody to GRASP65, followed by Alexa-594-conjugated donkey anti-mouse IgG (red channel), and Alexa-488-conjugated donkey anti-rabbit IgG (green channel). Nuclei were stained with DAPI (blue channel). Stained cells were examined by fluorescence microscopy. Merging of the images in the red, green, and blue channels generated the third image on each row; yellow indicates overlapping localization of the green and red channels. In A, arrows indicate colocalization at ER exit sites. In B, arrows indicate colocalization at accumulated tubules. In C, the arrow indicates colocalization in an accumulated tubule; empty arrowheads indicate tubules containing only Giantin; and filled arrowheads indicate tubules containing only GRASP65. Bar, 10 μm. (TIF) [file pone.0135260.s008.tif]

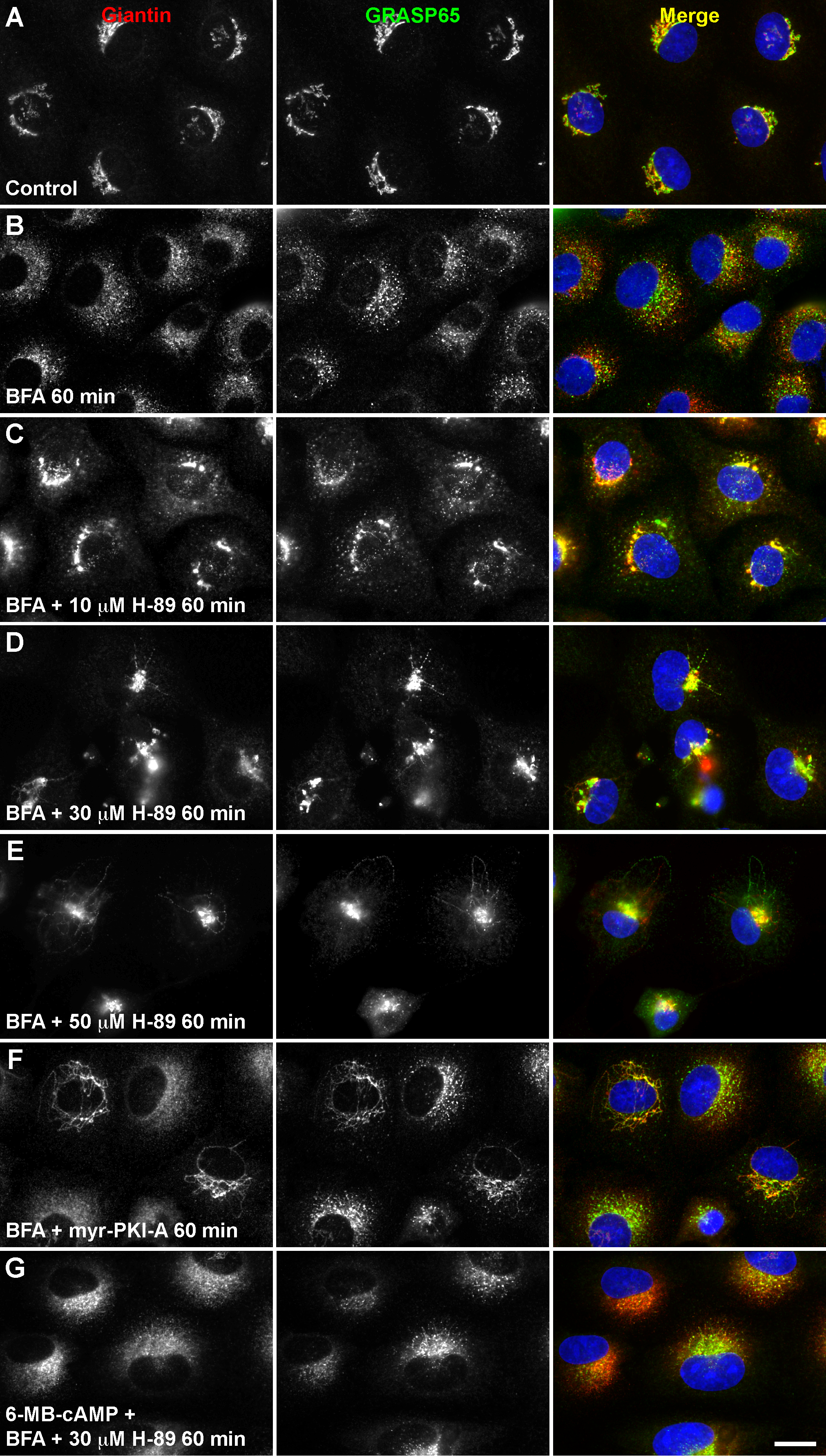

Supplement: S9 Fig — NRK cells were left untreated (A) or treated with 5 μg/ml BFA (B), or with BFA in conjunction with either H-89 at the indicated concentration (C-E) or 100 μM myr-PKI-A (F), or pre-incubated 5 min with 200 μM 6-MB-cAMP followed by addition of 5 μg/ml BFA and 30 μM H-89 (G), for the time indicated. Cells were fixed, permeabilized, immunolabeled with mouse monoclonal antibody to Giantin and rabbit antibody to GRASP65, followed by Alexa-594-conjugated donkey anti-mouse IgG (red channel), and Alexa-488-conjugated donkey anti-rabbit IgG (green channel). Nuclei were stained with DAPI (blue channel). Stained cells were examined by fluorescence microscopy. Merging of the images in the red, green, and blue channels generated the third image on each row; yellow indicates overlapping localization of the green and red channels. Bar, 10 μm. (TIF) [file pone.0135260.s009.tif]

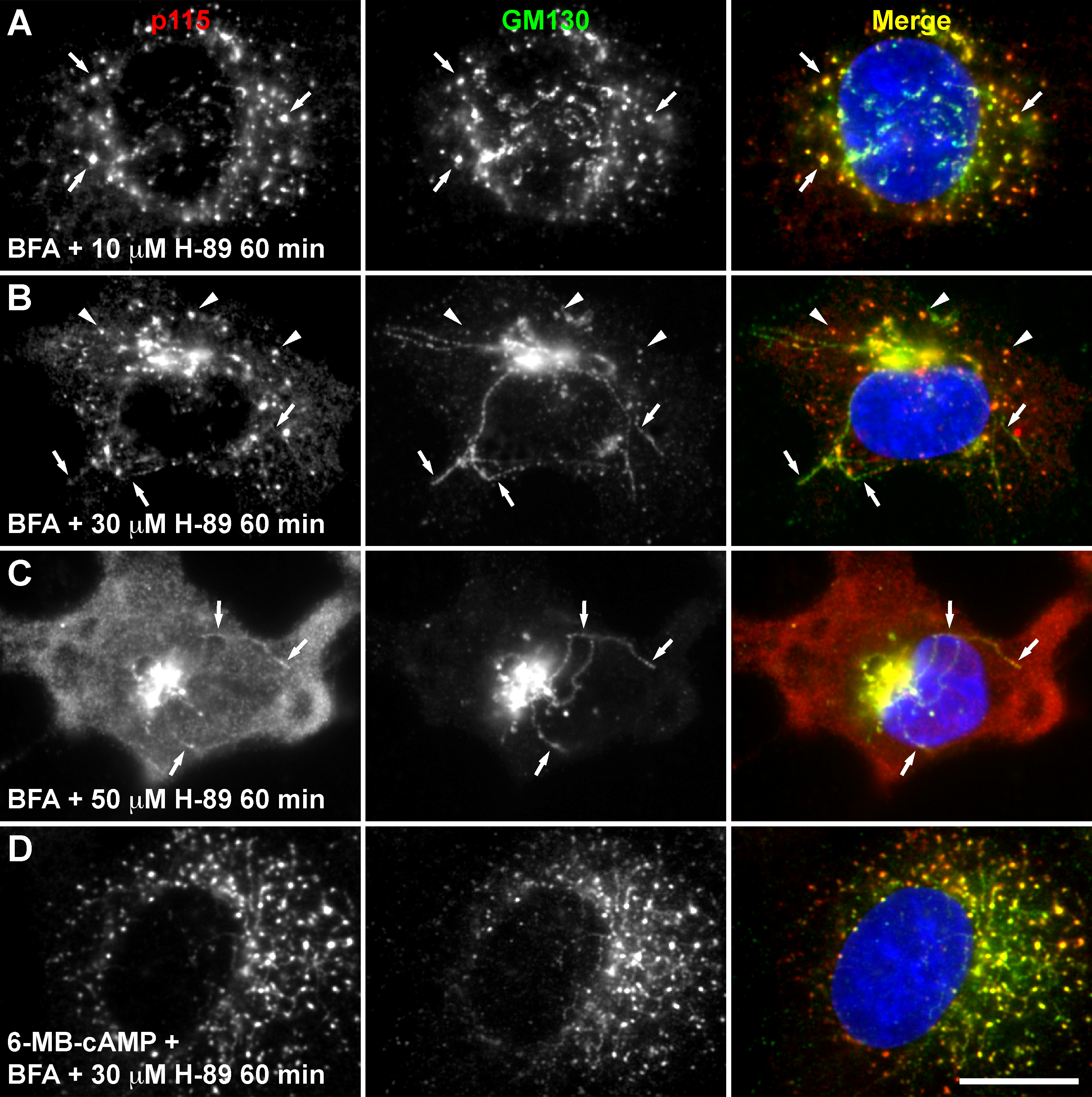

Supplement: S10 Fig — NRK cells were treated with 5 μg/ml BFA in conjunction with H-89 at the indicated concentration (A-C), or pre-incubated 5 min with 200 μM 6-MB-cAMP followed by addition of BFA and 30 μM H-89 (D), for the time indicated. Cells were fixed, permeabilized, immunolabeled with rabbit polyclonal antibody to p115 and mouse monoclonal antibody to GM130, followed by Alexa-594-conjugated donkey anti-rabbit IgG (red channel), and Alexa-488-conjugated donkey anti-mouse IgG (green channel). Nuclei were stained with DAPI (blue channel). Stained cells were examined by fluorescence microscopy. Merging of the images in the red, green, and blue channels generated the third image on each row; yellow indicates overlapping localization of the green and red channels. In A, arrows indicate colocalization at ER exit sites. In B, arrows indicate colocalization at accumulated tubules; and arrowheads indicate localization of p115 at ER exit sites. In C, arrows indicate colocalization in accumulated tubules. Bar, 10 μm. (TIF) [file pone.0135260.s010.tif]

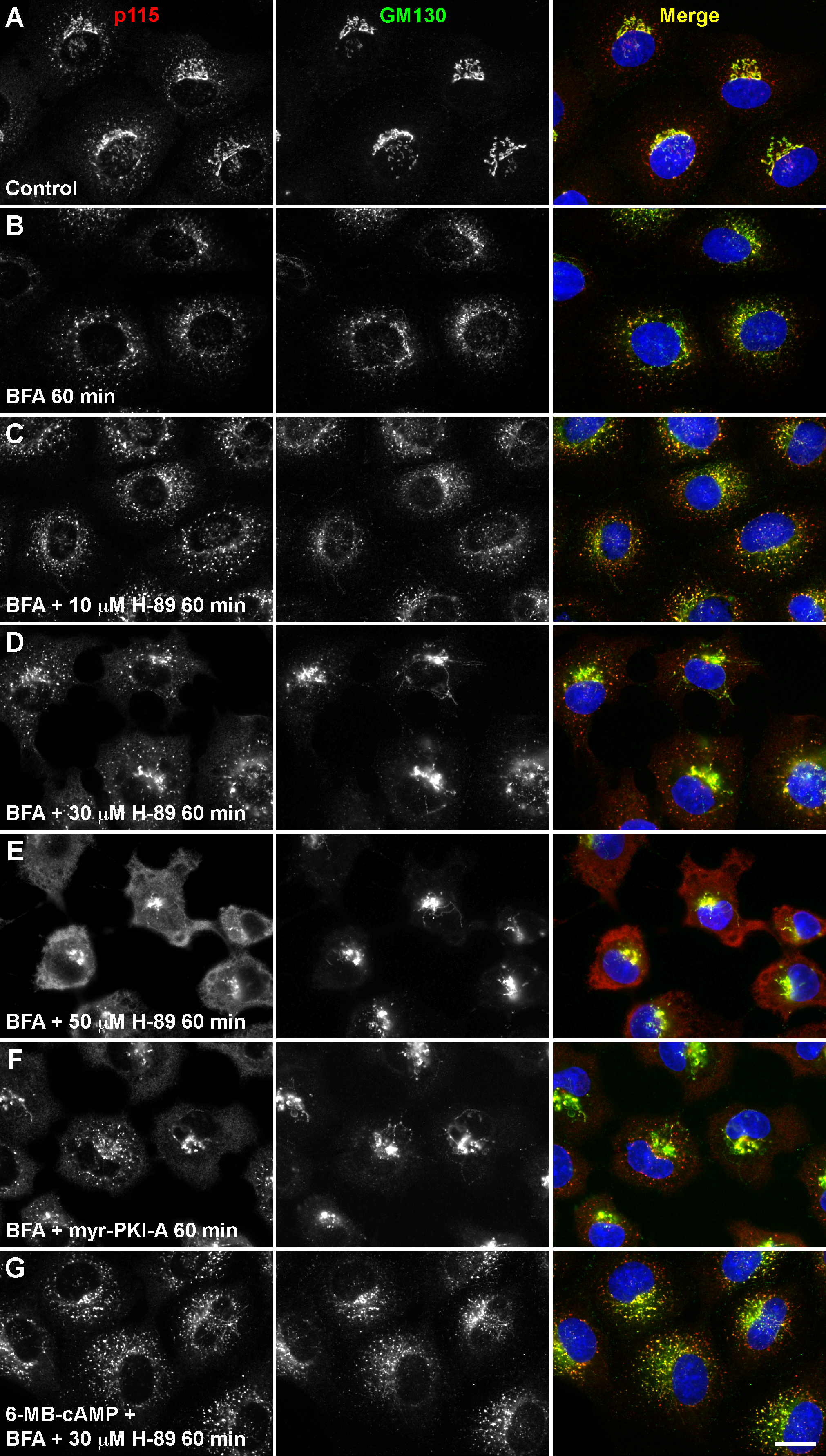

Supplement: S11 Fig — NRK cells were left untreated (A) or treated with 5 μg/ml BFA (B), or with BFA in conjunction with either H-89 at the indicated concentration (C-E) or 100 μM myr-PKI-A (F), or pre-incubated 5 min with 200 μM 6-MB-cAMP followed by addition of BFA and 30 μM H-89 (G), for the time indicated. Cells were fixed, permeabilized, immunolabeled with rabbit polyclonal antibody to p115 and mouse monoclonal antibody to GM130, followed by Alexa-594-conjugated donkey anti-rabbit IgG (red channel), and Alexa-488-conjugated donkey anti-mouse IgG (green channel). Nuclei were stained with DAPI (blue channel). Stained cells were examined by fluorescence microscopy. Merging of the images in the red, green, and blue channels generated the third image on each row; yellow indicates overlapping localization of the green and red channels. Bar, 10 μm. (TIF) [file pone.0135260.s011.tif]

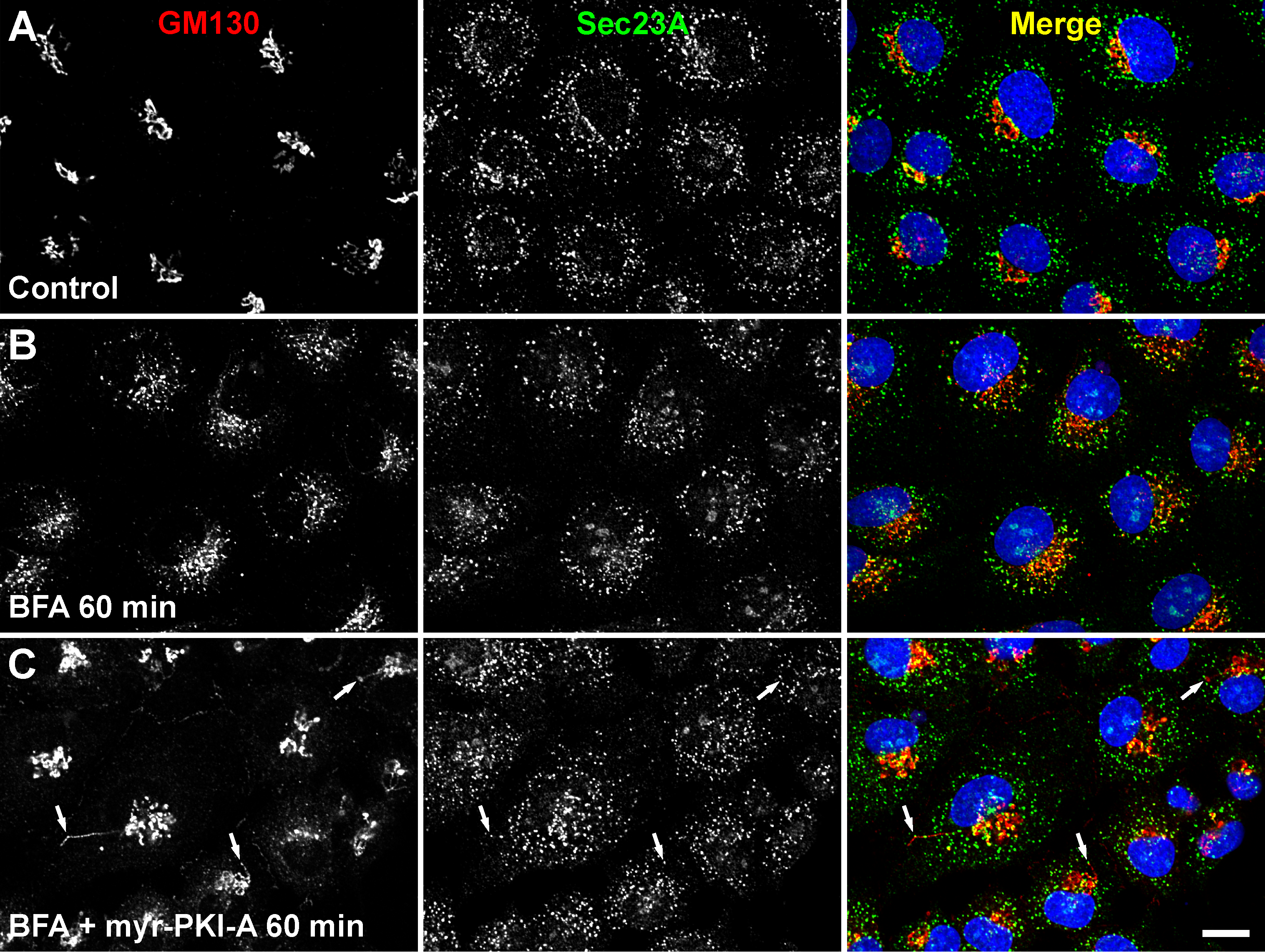

Supplement: S12 Fig — NRK cells were left untreated (A) or treated with 5 μg/ml BFA (B), or with BFA in conjunction with 100 μM myr-PKI-A (C), for the time indicated. Cells were fixed, permeabilized, immunolabeled with mouse monoclonal antibody to GM130 and rabbit antibody to Sec23A, followed by Alexa-594-conjugated donkey anti-mouse IgG (red channel), and Alexa-488-conjugated donkey anti-rabbit IgG (green channel). Nuclei were stained with DAPI (blue channel). Stained cells were examined by confocal fluorescence microscopy. Merging of the images in the red, green, and blue channels generated the third image on each row; yellow indicates overlapping localization of the green and red channels. In C, arrows indicate colocalization of tips of tubules or dilated portion of tubules with ER exit sites. Bar, 10 μm. (TIF) [file pone.0135260.s012.tif]

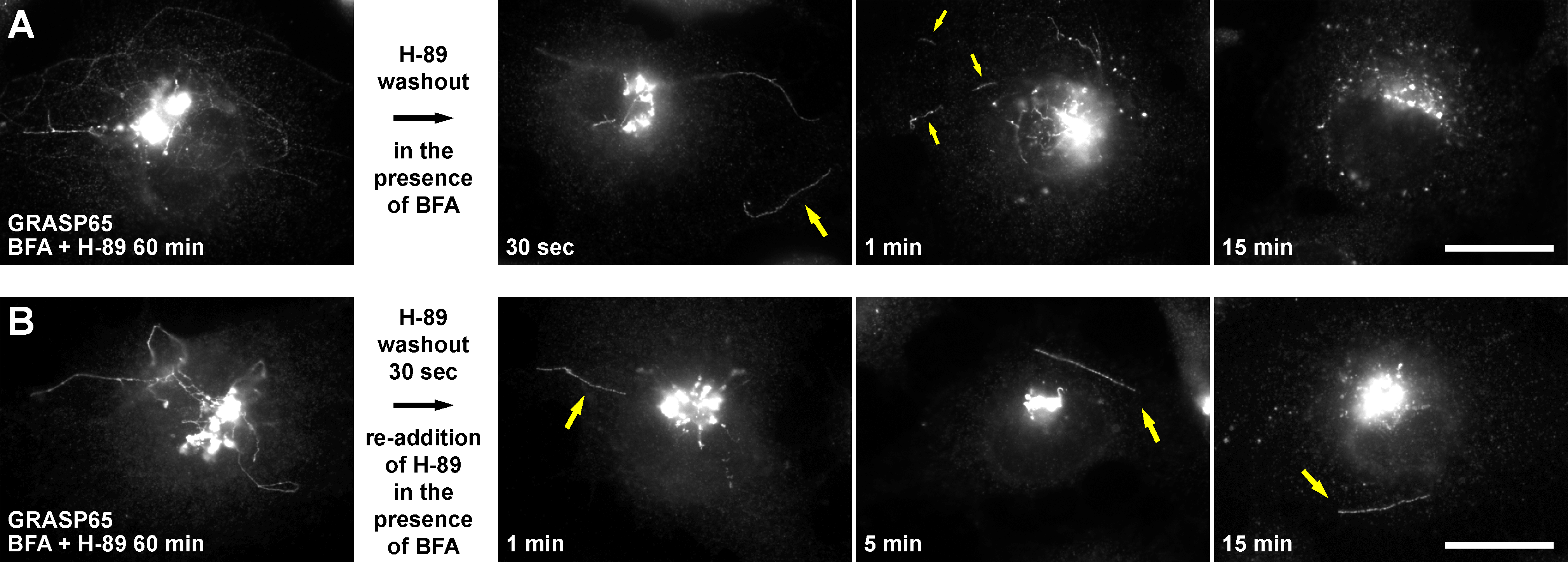

Supplement: S13 Fig — NRK cells were treated with 5 μg/ml BFA in conjunction with 30 μM H-89 (A-B). After 60 min, H-89 was either washed out in the presence of BFA, and cells were fixed at the times indicated (A), or H-89 was washed out for 30 sec in the presence of BFA, followed by re-addition of 30 μM H-89 in the presence of BFA, and cells were fixed at the times indicated (B). Cells were permeabilized and immunolabeled with rabbit polyclonal antibody to GRASP65, followed by Alexa-488-conjugated donkey anti-rabbit IgG. Stained cells were examined by fluorescence microscopy. Large arrows indicate large cut tubules. Small arrows indicate small cut tubules. Bar, 10 μm. (TIF) [file pone.0135260.s013.tif]
